# Genomic and insulin-mediated control of metabolic homeostasis by the mosquito ecdysone-induced gene E93

**DOI:** 10.1073/pnas.2511572122

**Published:** 2025-10-29

**Authors:** Xueli Wang, Danqian Geng, Kai Shi, Qi Qi, Xiangyang Lyu, Xiaomei Sun, Alexander S. Raikhel, Zhen Zou

**Affiliations:** ^a^State Key Laboratory of Animal Biodiversity Conservation and Integrated Pest Management, Institute of Zoology, Chinese Academy of Sciences, Beijing 100101, China; ^b^College of Life Sciences, University of Chinese Academy of Sciences, Beijing 101408, China; ^c^School of Life Science, Institute of Life Science and Green Development, Hebei University, Baoding 071002, China; ^d^Department of Entomology, University of California, Riverside, CA 92521; ^e^Institute of Integrative Genome Biology, University of California, Riverside, CA 92521

**Keywords:** reproduction, metabolic reprogramming, insulin, E93

## Abstract

Ecdysone-induced protein 93 (E93) is an adult specifier that governs insect pupal-adult conversion. It affects the reproductive transition in adult *Aedes aegypti* mosquitoes, the significant vectors of numerous devastating human diseases. Here, we show that E93 is essential for maintaining metabolic homeostasis during the reproductive cycle of mosquitoes. *E93* deficiency led to insufficient production of insulin-like peptide 3 (ILP3) from insulin-producing cells in the brain, resulting in reduced phosphorylation of protein kinase B (Akt), a key regulator in the insulin signaling pathway. This reduction facilitated the nuclear translocation of FoxO and enhanced the activity of glycogen synthase kinase 3β (GSK3β), which in turn respectively activated the transcription of genes encoding phosphoenolpyruvate carboxykinase (PEPCK) during gluconeogenesis and reduced glycogen synthesis. Further insulin rescue and the luciferase activity assays demonstrated that E93 directly inhibited *PEPCK* transcription. Ultimately, *E93*-depleted mosquitoes exhibited systemic metabolic reprogramming, characterized by the dysregulation of carbohydrate, lipid, and amino acid metabolism. Our findings establish that E93 orchestrates metabolic homeostasis by coordinating the insulin signaling cascade and directly regulating *PEPCK* expression, thus providing an intrinsic connection between endocrine signaling and E93-mediated reproduction in mosquitoes.

## Significance

Female mosquitoes are major disease vectors because they require blood feeding for reproduction. Metabolic events are synchronized with the reproductive cycles to meet their energy demands. Ecdysone-induced protein 93 (E93), a key factor governing insect metamorphosis and reproductive cyclicity, orchestrates metabolic homeostasis through a dual mechanism in mosquitoes. *E93* knockdown negatively affected insulin-like peptide 3 (ILP3) production, thereby inhibiting the activation of insulin signaling cascade. This resulted in reduced phosphorylation of protein kinase B (Akt) and glycogen synthase kinase 3β (GSK3β), and promoted FoxO nuclear translocation. Furthermore, E93 directly inhibited *PEPCK* expression. Accordingly, *E93* knockdown led to lower glycogen content and higher glucose levels. Our work provides insight into the metabolic regulation of disease-transmitting mosquitoes.

The blood-feeding behavior of mosquitoes is essential for vitellogenesis and ovarian development, facilitating the transmission of pathogens across different hosts. Hematophagous *Aedes aegypti* mosquitoes are vectors for transmitting devastating arboviral human diseases, including Zika, Yellow fever, and Dengue, and pose a tremendous threat to global public health (1). Given the absence of targeted treatments or effective vaccines, and the increasing resistance of mosquitoes to insecticides, controlling mosquito-borne diseases has become more challenging (2). Therefore, elucidating the molecular aspects of reproduction is crucial for developing effective means of controlling mosquito populations.

In most species, ranging from insects to mammals, reproductive events are accompanied by rapid metabolic changes in response to high energy requirements (3–6). The insect fat body acts analogously to vertebrates’ liver and adipose tissue, synchronizing energy storage and utilization with the insects’ reproductive process. Carbohydrates and lipids are the crucial components of the fat body, providing the principal energy fuel required for oocyte maturation, embryonic development, and offspring survival (7). Previous studies have reported that glucose metabolism was important during the embryogenesis of *Tribolium castaneum* and *A. aegypti* (8, 9). Similarly, lipid homeostasis is required for embryogenesis in *Bombyx mori* (10). The impairment of metabolic homeostasis in the fat body could lead to reproductive disorders. The disturbance of lipid mobilization reduces the reproductive capacity in various insects (11, 12). Thus, fine-tuning of metabolic flux via various regulatory factors is necessary for adapting to physiological demands during reproduction.

In the gonadotrophic cycle of *A. aegypti*, carbohydrate metabolism (CM) and lipid metabolism (LM) are governed by juvenile hormone (JH) and 20-hydroxyecdysone (20E). During the JH-mediated previtellogenic phase, the female fat body accumulates lipids and carbohydrates in preparation for blood meal-triggered events, providing energy reserves for ovarian development and egg maturation during the 20E-controlled vitellogenic period (4, 5). Additionally, nuclear receptors (NRs), neuropeptides, and microRNAs have been demonstrated to play a role in regulating metabolism in insects (3, 13, 14).

Insulin and insulin-like growth factors (IGF) signaling (IIS) serve as key endocrine regulators that orchestrate multiple physiological processes (15, 16). The crosstalk of IIS, JH, and 20E is vital in regulating metabolism during insect reproduction (17). Like *Drosophila melanogaster*, the genome of *A*. *aegypti* contains eight insulin-like peptides (ILPs), designated as ILP1 to ILP8 (18, 19). Insulin-producing cells (IPCs) in the brain are the primary source of ILPs. These cells can sense the circulating glucose levels and respond accordingly (20). ILPs are conserved in insects and act as activators to initiate the IIS pathway (21). Prior research has shown that knocking out each of the *ILP* genes by the CRISPR/Cas9 approach resulted in varying degrees of disruption in the CM and LM (14, 22). Serine/threonine protein kinase B (PKB/Akt) is a crucial effector of the IIS pathway (23). It is phosphorylated upon activation of the IIS pathway, thereby triggering the phosphorylation of its downstream substrates, such as FoxO and glycogen synthase kinase 3β (GSK3β). Subsequently, Akt-mediated phosphorylation of FoxO leads to its cytoplasmic retention, regulating the transcriptional expression of FoxO-dependent genes (24). Simultaneously, the phosphorylation of GSK3β results in the inactivation of its activity, thus promoting glycogen synthase activity and ultimately increasing glycogen levels (25).

Ecdysone-induced protein 93 (E93) belongs to the Pipsqueak transcription factor family. It is vital during insect development and reproduction (26, 27). Recently, E93 has also been determined to be tightly linked to insect metabolic homeostasis. Studies in *T. castaneum* have shown that loss of *E93* caused the failure of lipid accumulation in the fat bodies (28). The neuron-specific expression of E93 is related to regulating metabolic balance in *D. melanogaster* (29). Ligand-dependent corepressor (LCoR), the ortholog of E93 in mammals, plays an essential role in lipid homeostasis (30). In adult female *A. aegypti*, E93 is critical for regulating reproductive transition (31). Additionally, we have shown that changes in CM and LM were synchronized with mosquito reproduction (4, 5). However, the mechanistic understanding of E93’s role in metabolic homeostasis during the mosquito gonadotrophic cycle is lacking.

This study determined the metabolic status following *E93* depletion and investigated the underlying metabolic homeostasis during the reproductive cycle. Our findings demonstrate that E93 coordinates the metabolic balance by directly inhibiting the transcription of *PEPCK* and fine-tuning the IIS signaling cascade, thus responding to nutritional changes triggered by blood feeding. We provide evidence for a better understanding of mosquitoes’ reproductive processes, which is vital for developing strategies targeting mosquito control.

## Results

### Knockdown of *E93* Triggers the Metabolic Reprogramming in Female Mosquitoes.

Our previous studies have shown that genes associated with LM and CM exhibited differential expression at 36 h post–blood meal (PBM) in mosquitoes following *E93* RNAi (iE93) compared to *EGFP* RNAi (iEGFP) (31). We performed the transcriptomic analysis of gene expression and Kyoto Encyclopedia of Genes and Genomes (KEGG) analysis to further understand the effect of E93 on the metabolic processes of LM and CM. Our findings revealed that most genes involved in fatty acid degradation, fatty acid biosynthesis, glycogen/trehalose metabolism, glycolysis/gluconeogenesis, citrate cycle, and the pentose phosphate pathway were significantly downregulated in response to the *E93* knockdown (*SI Appendix*, Fig. S1*A* and Dataset S1). Additionally, we conducted the real-time qPCR to confirm the consistency with the transcriptomic data (*SI Appendix*, Fig. S1*B*). These results indicate that the metabolic homeostasis of mosquitoes is drastically affected after the *E93* knockdown.

**Fig. 1.**
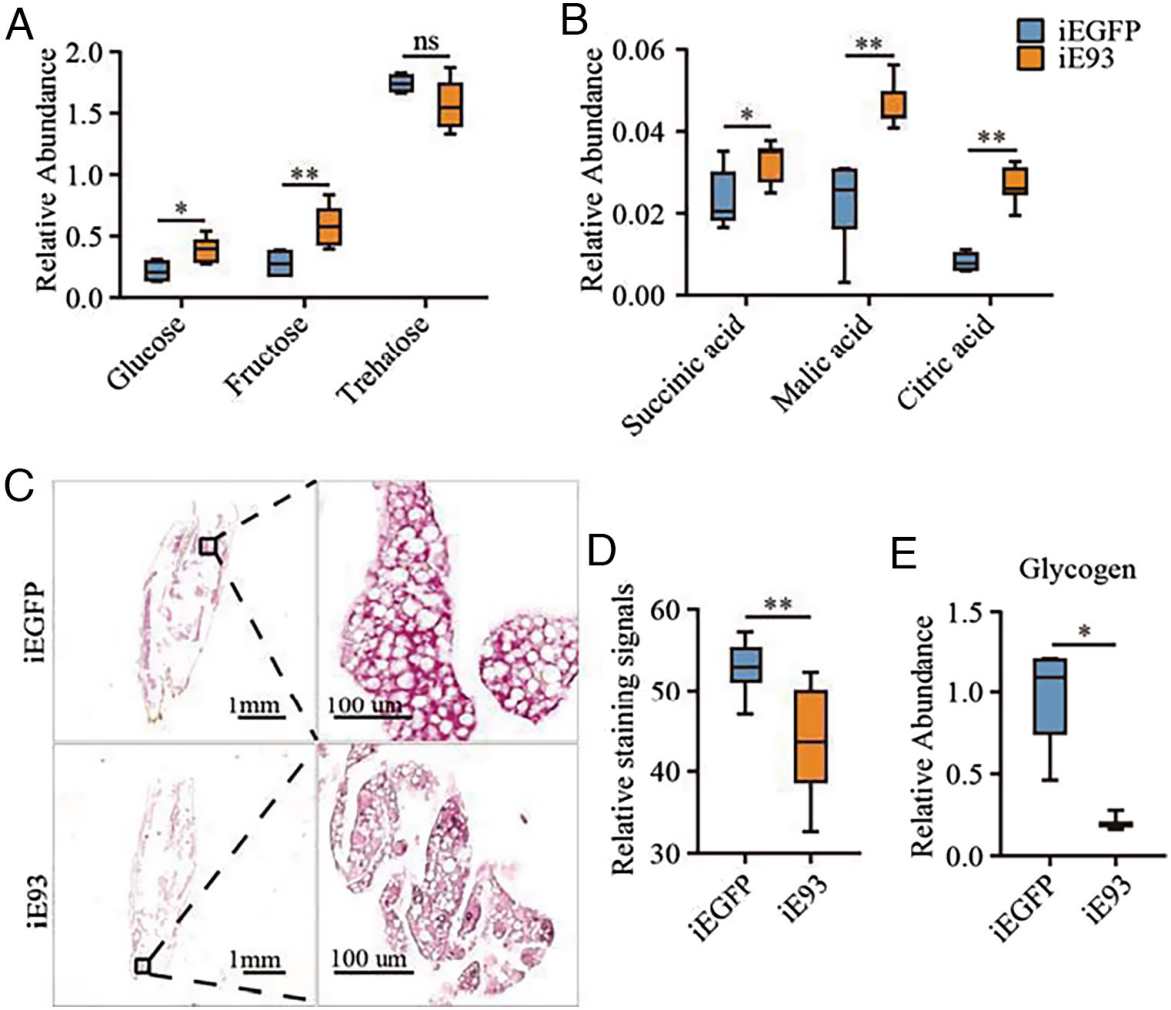
*E93* knockdown leads to metabolic defects in mosquitoes. (*A* and *B*) GC/MS was conducted to detect the relative levels of glucose, fructose, and trehalose (*A*), and the major metabolites, including succinic acid, malic acid, and citric acid of the TCA cycle (*B*), in the fat bodies of iEGFP and iE93 mosquitoes. *n* ≥ 4. (*C*) Glycogen amounts were observed by PAS staining. Scale bars are included in each figure. (*D*) Quantitative analysis of the staining intensity of glycogen from (*C*). *n* = 11 and 7 for iEGFP and iE93, respectively. (*E*) Endogenous glycogen content in iE93 mosquitoes and controls. *n* ≥ 3. Box plots in (*A* and *B*) and (*D* and *E*) display the median, lower, and upper quartiles. Error bars show the minimum and maximum values. Statistical significance of two-group comparisons was determined by a two-tailed unpaired *t* test with Welch’s correction for (*A* and *B*), and a Mann–Whitney *U* test for (*D* and *E*). *P < 0.05, **P < 0.01. ns, not significant.

To further elucidate the role of E93 in metabolism, we employed gas chromatography/mass spectrometry (GC/MS) to compare the metabolic profiles between iEGFP and iE93 mosquitoes. The circulating glucose and fructose levels were significantly elevated in iE93 mosquitoes compared to those in iEGFP controls. In contrast, trehalose levels remained unchanged between these two groups (Fig. 1*A*). Furthermore, key intermediates in the citrate cycle, including succinic acid, malic acid, and citric acid, also increased in iE93 mosquitoes (Fig. 1*B*). We performed Periodic acid/Schiff (PAS) staining to visualize the glycogen content in the fat bodies, which showed a significant reduction in the PAS-positive signal in iE93 mosquitoes (Fig. 1*C*). Next, using the HALO software, we conducted the statistical analysis for PAS signal of the stained iEGFP and iE93 mosquitoes. Consistently, iE93 mosquitoes exhibited a notable decrease in glycogen staining signals (Fig. 1*D*). Furthermore, the colorimetric measurement of glycogen levels using a glucose assay kit aligned with the glycogen staining outcomes, showing a substantial reduction following the *E93* knockdown (Fig. 1*E*).

We assessed whether changes in lipid content that correspond to the LM enzymes occur in the fat body of iE93 mosquitoes. There was a marked accumulation of lipid droplets (LDs) in mosquitoes treated with dsE93 (double-stranded RNA of *E93*), compared to those treated with dsEGFP (*SI Appendix*, Fig. S2*A*). The relative size of LDs was significantly larger in iE93 females than in the iEGFP control (*SI Appendix*, Fig. S2*B*). Triacylglycerol (TAG), the predominant lipid storage form, increased dramatically following the *E93* knockdown, exceeding levels in the control mosquitoes (*SI Appendix*, Fig. S2*C*). Furthermore, free fatty acids (FFAs), including saturated FFAs (C16:0 and C18:0) and unsaturated FFAs (C16:1, C18:1, and C18:2), exhibited a phenotype similar to TAG in iE93 mosquitoes. Likewise, amino acids such as alanine, glutamine, proline, threonine, and tyrosine were notably higher in iE93 mosquitoes than in controls (*SI Appendix*, Fig. S2 *D* and *E*). These findings suggest that the knockdown of *E93* disrupts the normal metabolism of carbohydrates, lipids, and amino acids, indicating a comprehensive metabolic dysregulation in response to *E93* depletion in *A. aegypti* mosquitoes.

### *E93* Knockdown Changes the Chromatin Accessibility across the Genome.

**Fig. 2.**
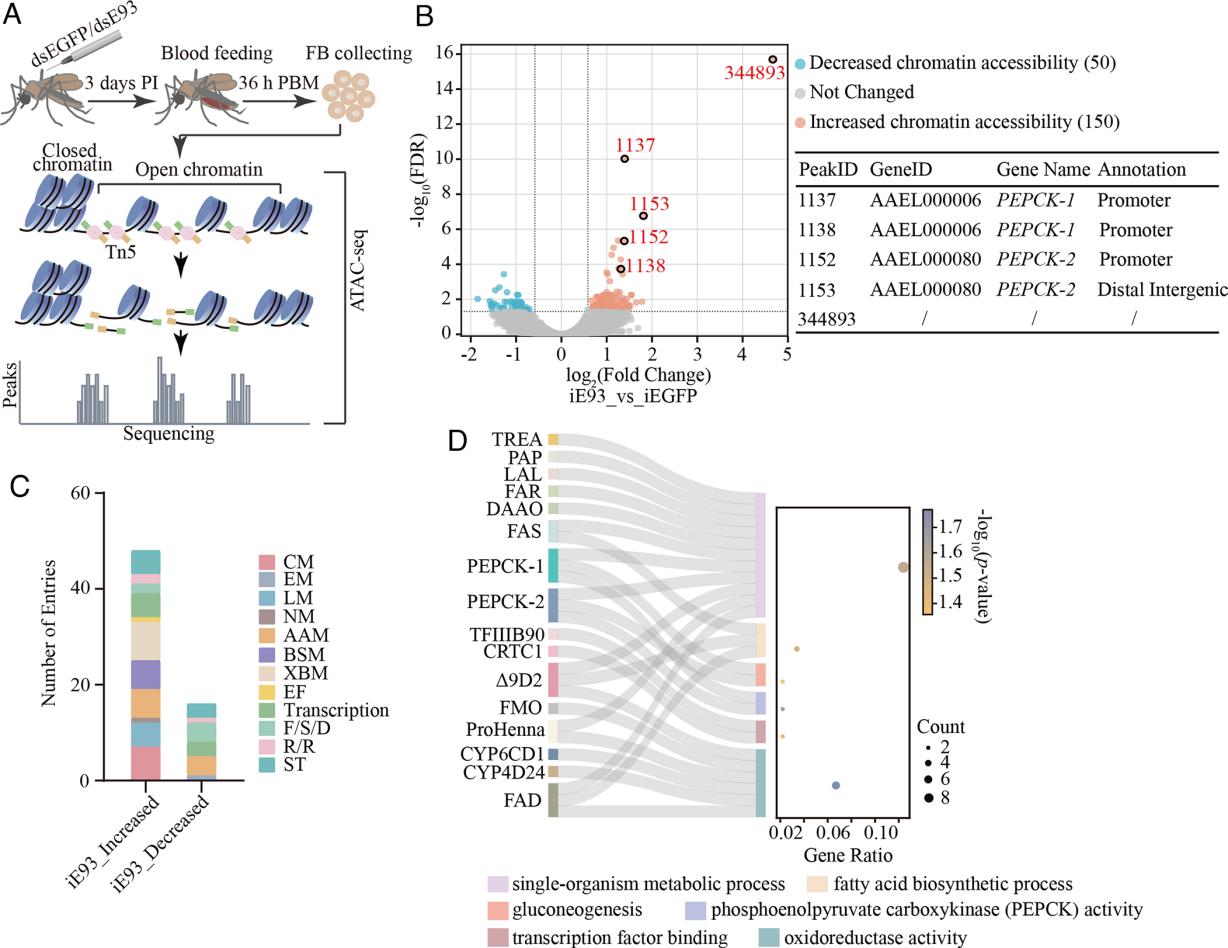
E93 regulates chromatin accessibility. (*A*) Schematic diagram of the ATAC-seq assay. Tn5 is the transposase used in ATAC-seq. (*B*) The volcano plot displays differential chromatin accessible peaks in the fat bodies of iEGFP and iE93 mosquitoes. Decreased, unchanged, and increased chromatin accessible peaks are marked with blue, gray, and red dots, respectively. Significantly increased chromatin accessible peaks with large fold change are highlighted with a black circle, and their corresponding annotations are listed in the right table. (*C*) Functional distribution of target genes corresponding to iE93-increased and -decreased peaks. CM, carbohydrate metabolism; EM, energy metabolism; LM, lipid metabolism; NM, nucleotide metabolism; AAM, amino acid metabolism; BSM, biosynthesis of other secondary metabolites; XBM, xenobiotics biodegradation and metabolism; EF, enzyme families; F/S/D, folding, sorting, and degradation; R/R, replication and repair; ST, signal transduction. (*D*) Sankey and dot plot reveals the target genes corresponding to the obviously increased peaks after *E93* knockdown. Columns in the Sankey plot represent gene names (on the *Left*) and the corresponding GO terms (on the *Right*). Terms mentioned in the GO analysis are shown at the *Bottom* of the plot. Dot sizes indicate gene counts in each term, and the colors show the *P-*value (*P* < 0.05).

ATAC-seq (Assay for Transposase-Accessible Chromatin with high-throughput sequencing) has been utilized to map the global landscape of chromatin accessibility across the genome, providing valuable insights into gene regulatory mechanisms (32). E93 has been shown to modulate gene expression by altering chromatin accessibility during *D. melanogaster* development (33, 34). We utilized ATAC-seq to identify potential target genes affected by E93 in adult female mosquitoes during reproduction. The dsRNAs of *E93* and *EGFP* were prepared and injected into mosquitoes within 24 h postemergence. After 3 d of recovery, injected mosquitoes were given a blood meal. Then, fat bodies were collected at 36 h PBM to perform library construction and ATAC-seq analysis (Fig. 2*A*).

Peak regions identified by the ATAC-seq were annotated according to their proximity to various gene functional elements in the genome, including promoters, 3’-untranslated regions (3’-UTRs), 5’-untranslated regions (5’-UTRs), exons, introns, and distal intergenic regions. Notably, over 65% of the identified peaks were in the distal intergenic regions, while more than 14% were found in the promoter regions (*SI Appendix*, Fig. S3*A*). These peaks were then analyzed for differential accessibility using the R package DiffBind with a cutoff of a fold change (FC) greater than 1.5 or less than 0.67 and a false discovery rate (FDR) of less than 0.05. A total of 200 differentially accessible regions (DARs) were identified, with 150 showing increased accessibility (indicating open chromatin) and 50 showing decreased accessibility (indicating closed chromatin) (*SI Appendix*, Fig. S3*B* and Dataset S2). These data suggest that most genes are likely activated following *E93* knockdown, which is in accordance with the earlier transcriptomic results, showing more iE93-upregulated genes than iE93-downregulated ones at 36 h PBM (31).

To visually distinguish the differential peaks between iEGFP and iE93 mosquitoes, a volcano plot was generated using the ATAC-seq signals with an FDR less than 1. The plot displayed the changes in total peaks, including the increased chromatin accessibility (FC > 1.5 and FDR < 0.05), the decreased chromatin accessibility (FC < 0.67 and FDR < 0.05), and those that were not statistically significant changes in chromatin accessibility (Fig. 2*B*). Peak 344893 (PeakID used in this work, Dataset S3) demonstrated the most substantial increase in chromatin accessibility in mosquitoes with *E93* knockdown. However, it lacks annotated information. Then, we investigated other peaks with significant increases, including peaks 1137 and 1153. In the annotation analysis of the peak regions, peak 1137 was identified as being in the promoter region of the gene encoding phosphoenolpyruvate carboxykinase-1 (PEPCK-1, AAEL000006), while peak 1153 was found in the distal intergenic region of the *PEPCK-2* gene (AAEL000080). Peaks 1152 and 1138 were assigned to the promoter regions of both *PEPCK-2* and *PEPCK-1* genes, respectively (Fig. 2*B*). PEPCKs, the key enzymes in the gluconeogenesis pathway, played a crucial role in glucose production in vivo (35). Based on these observations, we propose that *E93* knockdown could lead to the recruitment of regulatory factors to the promoter or distal intergenic region, thereby modulating the expression of *PEPCK-1* and *PEPCK-2*.

Next, we explored the functional classification of target genes associated with the corresponding DARs through the KEGG analysis. Results revealed remarkable changes in the functional distributions of genes upon *E93* knockdown. Notably, genes related to CM and LM were predominantly enriched in iE93-increased clusters compared to iE93-decreased clusters (Fig. 2*C*). These findings further confirmed the importance of E93 in metabolism regulation. To elucidate the regulatory mechanism of E93 on CM and LM, we performed Sankey and dot analyses on the target genes corresponding to iE93-increased peaks (*SI Appendix*, Tables S1 and S2). Results demonstrated that the target genes were primarily involved in the biological processes of single-organism metabolism, fatty acid biosynthesis, and gluconeogenesis. Furthermore, the molecular function of these genes was mainly associated with PEPCK activity, transcription factor binding, and oxidoreductase activity (Fig. 2*D*).

### *E93* Knockdown Enhances the Expression and Chromatin Accessibility of *PEPCK* Genes.

**Fig. 3.**
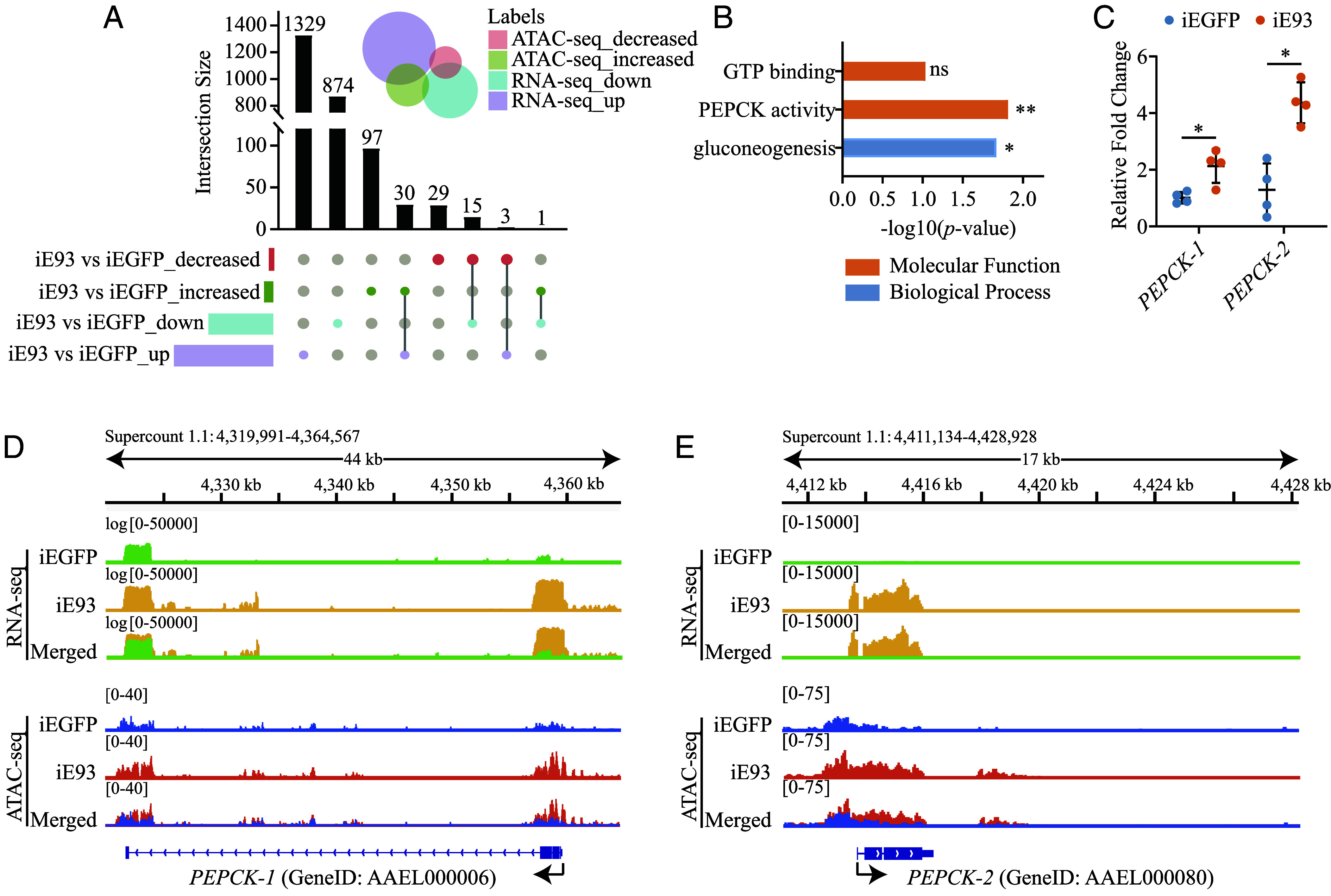
Integrative analysis of the RNA-seq and ATAC-seq in *A. aegypti* following *E93* knockdown. (*A*) Venn diagram and UpSet plot present the uniquely and commonly regulated genes in the transcriptome and ATAC-seq data. (*B*) GO analysis of the shared genes from ATAC-seq-increased and RNA-seq-up datasets after *E93* knockdown. The DAVID web server was used in this analysis, and a two-sided Fisher’s exact test was used to determine the *P*-value. (*C*) Expression of *PEPCK-1* and *PEPCK-2* in iEGFP and iE93 mosquitoes (*n* = 4). A two-tailed unpaired *t* test determined statistical significance. (*D* and *E*) Visualization of the read coverage and chromatin accessibility of two *PEPCK* genes using IGV. *P < 0.05, **P < 0.01, ns, not significant.

RNA-seq of iE93 mosquitoes at 36 h PBM identified 2,252 differentially expressed genes (DEGs), with 1,362 upregulated and 890 downregulated (31). To further elucidate gene expression regulation in the absence of *E93*, an integrative analysis of RNA-seq and ATAC-seq was performed. The UpSet plot revealed 49 genes correlated between transcriptional alterations and differentially accessible chromatin regions. Among these, 30 genes exhibited upregulated mRNA abundance and increased chromatin accessibility, while 15 showed downregulated mRNA levels and reduced chromatin accessibility (Fig. 3*A* and Dataset S4).

Subsequently, the 30 upregulated genes from Fig. 3*A* underwent GO analysis. Notably, GO terms related to PEPCK activity and gluconeogenesis were significantly enriched in iE93 mosquitoes (Fig. 3*B* and *SI Appendix*, Table S3). The mRNA levels of *PEPCK-1* and *PEPCK-2* were elevated in iE93 mosquitoes compared to those in iEGFP controls, as confirmed by qPCR (Fig. 3*C*). The IGV software was used to analyze the RNA-seq and ATAC-seq data, visualizing the read coverage and peak changes of *PEPCK-1* and *PEPCK-2*. iE93 mosquitoes showed higher read coverage in the coding regions and increased peaks in the promoter regions of these two genes (Fig. 3 *D* and *E*). Moreover, *PEPCK-2* exhibited additional increased peaks in the distal intergenic region. Overall, these data suggest that the upregulated expression of *PEPCK* genes leads to the activation of gluconeogenesis, which subsequently increases glucose levels in the fat body of mosquitoes subjected to *E93* depletion.

### FoxO Activates the Transcription of *PEPCK* in *E93*-Depleted Mosquitoes.

**Fig. 4.**
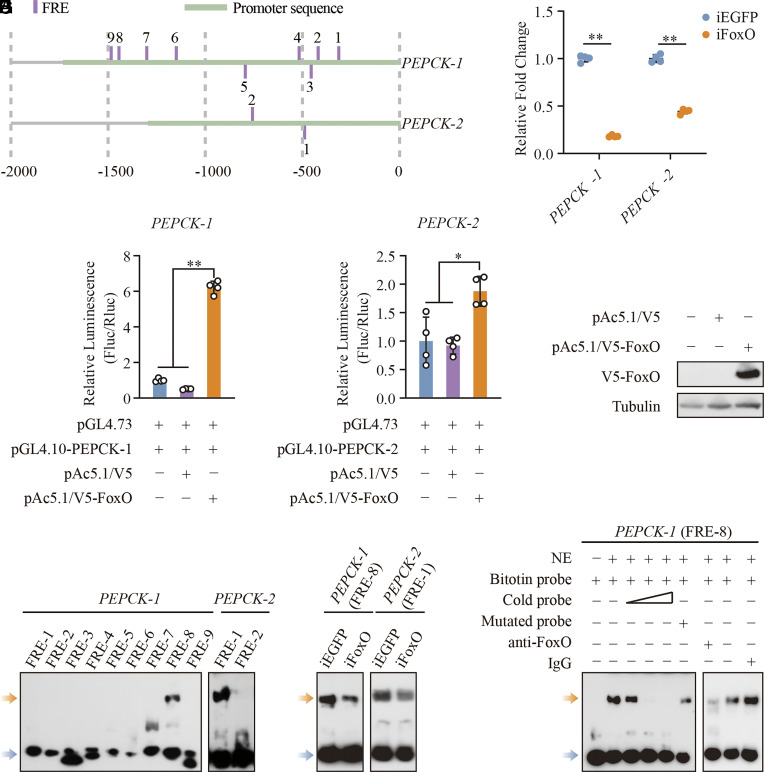
FoxO upregulates *PEPCK* expression. (*A*) A schematic diagram showing the genomic loci of predicted FREs in *PEPCK* promoters. (*B*) Expression of two *PEPCK* genes following *FoxO* knockdown (*n* = 4). Effects of overexpressed V5-FoxO on *PEPCK-1* (*C*) and *PEPCK-2* (*D*) promoters detected by dual-luciferase reporter assay. *n* = 4. (*E*) Western blot detected the overexpression of V5-FoxO protein in Aag2 cells. Tubulin was used as the loading control. (*F*) EMSA showing the binding profile between FREs located in two *PEPCK* promoters and the NEs from the whole body of female mosquitoes. (*G*) Effects of *FoxO* knockdown on the binding of FRE-8 probe of *PEPCK-1* and FRE-1 probe of *PEPCK-2* with NEs. (*H*) EMSA illustrating the binding between NEs isolated from WT mosquitoes and FRE-8 in *PEPCK-1*. Yellow and blue arrows in (*F*–*H*) represent the shift bands and free probes, respectively. Statistical significance was determined by a two-tailed unpaired *t* test with Welch’s correction for (*B*) and one-way ANOVA with Dunnett’s T3 test for (*C* and *D*). Data represent mean ± SD. *P < 0.05, **P < 0.01.

The accessibility of chromatin in the promoter region is pivotal for regulating gene expression, as it enables the specific binding of transcription factors to the promoter and thus influences the genes’ transcriptional activity. The promoter regions of the *PEPCK-1* and *PEPCK-2* genes were open in iE93 mosquitoes. Based on the areas of open chromatin within the promoters, sequences of *PEPCK-1* (from −1,727 to +1) and *PEPCK-2* (from −1,290 to +1) were selected for the following JASPAR analysis, with relative scores of up to 90%. The FoxO response element (FRE) was identified within the promoter sequences of both genes, with 9 FREs (numbered 1 to 9) in the promoter of *PEPCK-1* and 2 FREs (numbered 1 and 2) in the promoter of *PEPCK-2*, respectively (Fig. 4*A*).

FoxO maintains a metabolic balance across various cells and tissues. It is a crucial regulator of gluconeogenesis by modulating PEPCK activity (36, 37). Therefore, we measured the *PEPCK* expression in mosquitoes treated with dsEGFP and dsFoxO. Notably, *FoxO* knockdown reduced the mRNA levels of both *PEPCK-1* and *PEPCK-2* (Fig. 4*B*). We next explored the transcriptional regulation of *PEPCK* by FoxO in vitro through dual-luciferase reporter assays. When FoxO was overexpressed in Aag2 cells, the transcriptional activity of *PEPCK-1* was significantly enhanced (12.28- or 6.21-fold increase compared to negative control cells that cotransfected with the reporter vectors and pAc5.1/V5 empty vector or transfected with the reporter vectors alone, respectively) (Fig. 4*C*). This enhancement was greater than that observed for *PEPCK-2* (2.04- or 1.88-fold increase, respectively) (Fig. 4*D*). Furthermore, Western blot analysis confirmed the overexpression of V5-tagged FoxO protein in pAc5.1/V5-FoxO transfected cells using an anti-V5 antibody. In contrast, the V5-FoxO protein was undetectable in two negative control cells (Fig. 4*E*), suggesting the increased luciferase activity in Fig. 4 *C* and *D* was attributed to the overexpressed FoxO protein.

To confirm the direct binding of FoxO to FRE located in the promoters of the two *PEPCK* genes, we conducted an electrophoretic mobility shift assay (EMSA) using nuclear extracts (NE) derived from whole mosquitoes at 72 h posteclosion (PE) and different FRE probes. Only the FRE-8 probe in the *PEPCK-1* promoter and the FRE-1 probe in the *PEPCK-2* promoter formed the DNA–protein complexes with NEs (Fig. 4*F*). Therefore, we used these two probes to assess their binding to the FoxO protein. To ensure that the binding specificity of the selected probes was associated with FoxO, NEs from iFoxO and iEGFP whole mosquitoes were used to perform EMSA. The intensity of the specific binding complexes was reduced when NEs from iFoxO mosquitoes were incubated with a biotin-labeled FRE-8 (*PEPCK-1*) probe. In contrast, the band’s intensity between the same NEs and FRE-1 (*PEPCK-2*) probe did not show apparent changes (Fig. 4*G*). These results suggest that FoxO is involved in the specific binding with the FRE-8 probe from *PEPCK-1*, rather than the FRE-1 probe from *PEPCK-2*. Considering the stringency of our initial JASPAR search, a more relaxed filtering threshold is necessary to identify the potential FRE in the *PEPCK-2* promoter.

To validate whether FoxO is directly bound to the *PEPCK-1* promoter, we conducted the EMSA using NEs from wild-type whole mosquitoes and a biotin-labeled FRE-8 (*PEPCK-1*) probe. Consistent with Fig. 4*F*, the specific DNA–protein binding band was detected after the incubation of NEs with the biotin-labeled FRE-8 probe, but there was no shift band in the absence of NEs. This specific band gradually disappeared after increasing amounts of the unlabeled specific probe (10×, 50×, or 100× molar excess) were added to the binding reactions. However, a notable band shift was observed after incubation with a 100× molar excess of unlabeled probe, in which the core FoxO binding nucleotides were mutated. This implies the importance of core FoxO binding sites in forming binding complexes. In addition, the shift band dramatically reduced when NEs were preincubated with anti-FoxO polyclonal antibody, but not the IgG antibody. These observations proved the presence of FoxO in the NE-DNA binding complexes (Fig. 4*H*). Thus, it appears that following *E93* knockdown, FoxO activates the transcription of *PEPCK-1* by directly binding to the FRE within the *PEPCK-1* promoter of *A. aegypti* female mosquitoes.

### RNAi-Mediated *E93* Knockdown Leads to the Nuclear Translocation of FoxO.

**Fig. 5.**
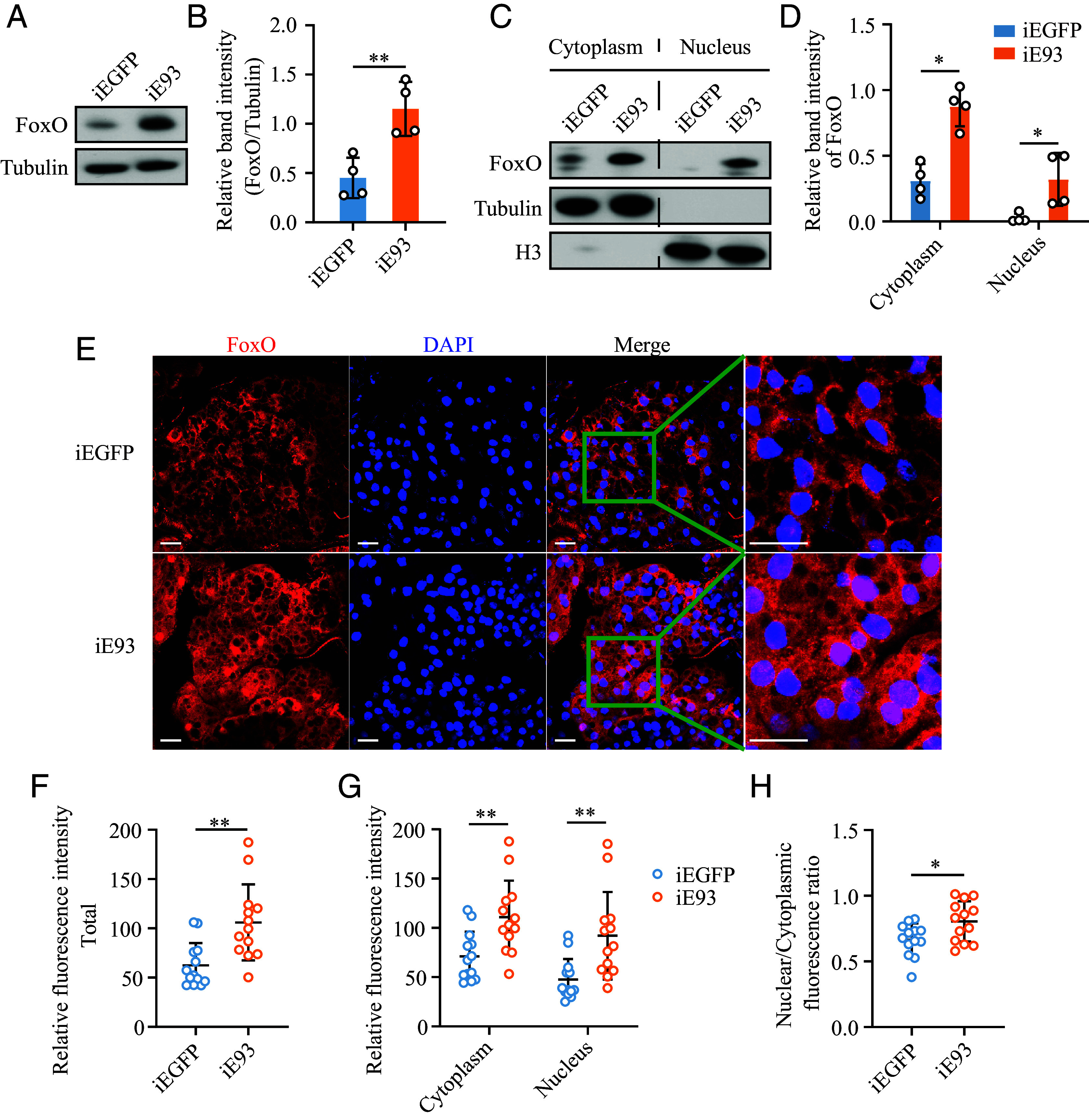
*E93* knockdown facilitates nuclear import of FoxO. (*A*) Effects of *E93* knockdown on FoxO protein levels in the fat body. Tubulin was used as the loading control. (*B*) The relative band intensity of panel (*A*) was digitized by ImageJ. (*C*) Western blot displayed the impact of *E93* knockdown on the levels of FoxO proteins in the cytoplasm and nucleus. Tubulin and H3 antibodies were used as the loading control for the cytoplasm and nucleus, respectively. (*D*) The relative band intensity of panel (*C*) was digitized using ImageJ. (*E*) Immunofluorescence detected the distribution of FoxO proteins (red) in iEGFP and iE93 mosquitoes. Nucleus (blue) was stained with Hoechst 33342. The area in the green boxes is magnified to the right of each group. Scale bar: 20 µm. The relative fluorescence intensities of the FoxO signals shown in panel (*E*) were quantified using ImageJ software for total (*F*), cytoplasmic and nuclear (*G*) compartments. (*H*) Effects of *E93* knockdown on the nuclear import ratio of FoxO. For (*F*–*H*), *n* = 13. The Mann–Whitney *U* test was used in the statistical analysis of (*B*, *D*, and *F*–*H*). Data represent mean ± SD. *P < 0.05, **P < 0.01.

Given that nuclear translocation is the crucial process for FoxO’s ability to regulate gene expression (38), the subcellular distribution of FoxO in iE93 mosquitoes was compared with that in iEGFP mosquitoes. First, we detected the differences in FoxO protein levels between mosquitoes subjected to *E93* and *EGFP* RNAi. The iE93 mosquitoes exhibited a higher abundance of FoxO protein compared to the iEGFP controls (Fig. 5 *A* and *B*). The unstimulated FoxO is phosphorylated and mainly located in the cytoplasm. However, in response to cellular stress or specific signaling events, FoxO is dephosphorylated and then translocated into the nucleus (38). To determine whether the increased abundance of FoxO protein in iE93 mosquitoes was attributed to its elevated levels in the nucleus or in the cytoplasm, we extracted the nuclear and cytoplasmic proteins from the fat bodies of iE93 mosquitoes to carry out the Western blot analysis. The results showed a 1.82-fold and 3.13-fold upregulation of the FoxO protein in the cytoplasm and nucleus, respectively, following depletion of *E93*. These observations confirm that although *E93* deficiency results in an increased level of FoxO both in the cytoplasm and nucleus, it exhibits a more pronounced accumulation in the nucleus (Fig. 5 *C* and *D*).

To further visualize the subcellular distribution of FoxO protein, we subjected the fat body tissue to an immunofluorescence examination. Consistent with the Western blot results, the immunofluorescence revealed that FoxO was primarily localized in the cytoplasm of the iEGFP mosquitoes, with almost undetectable signals in the nucleus. However, in iE93 mosquitoes, FoxO signals increased significantly in both the cytoplasm and the nucleus (Fig. 5*E*). ImageJ analysis showed that the total fluorescence intensity of FoxO in the fat bodies of iE93 mosquitoes was 47.29% higher than that of iEGFP mosquitoes (Fig. 5*F*). Notably, the fluorescence intensity of FoxO increased by 35.8% in the cytoplasm and 44.47% in the nucleus, respectively (Fig. 5*G*). We evaluated the ratio of nuclear to cytoplasmic fluorescence intensity of FoxO protein after the treatment with dsE93 or dsEGFP. As shown in Fig. 5*H*, iE93 mosquitoes displayed a 13.95% increase in the nuclear import efficiency compared to iEGFP controls. This strongly suggests the critical role of E93 in promoting the translocation of FoxO from the cytoplasm to the nucleus, which in turn leads to the upregulated expression of *PEPCK* genes.

### E93 Is Required for Modulating Insulin-Like Peptide 3 (ILP3) Levels in the IPCs.

**Fig. 6.**
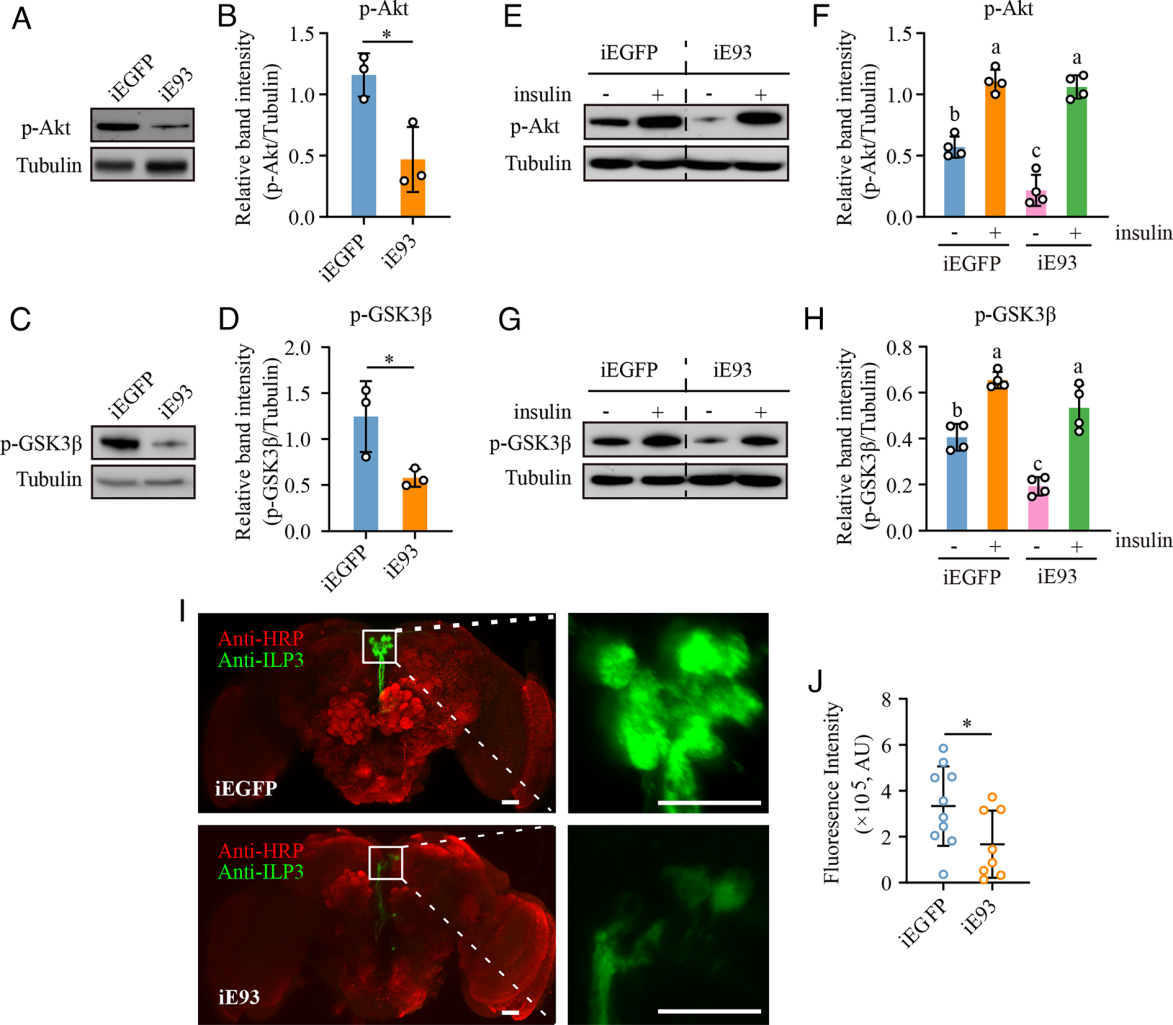
E93 regulates the insulin signaling pathway. (*A*) Western blot detected p-Akt levels in iE93 and iEGFP mosquitoes. (*B*) ImageJ digitized the relative band intensity of panel (*A*). (*C*) Western blot detected the p-GSK3β levels in iE93 and iEGFP mosquitoes. (*D*) The relative band intensity of panel (*C*) was digitized by ImageJ. (*E*) Effects of insulin supplement on p-Akt levels. (*F*) The relative band intensity of panel (*E*) was digitized by ImageJ. (*G*) Effects of insulin supplement on the p-GSK3β levels. (*H*) The relative band intensity of panel (*G*) was digitized using ImageJ. (*I*) Immunofluorescence of the endogenous ILP3 (green) in iEGFP and iE93 mosquitoes’ brains. Neural cells (red) in the brain were stained with anti-HRP antibody. The area in the white boxes is magnified on the right of each group. Scale bar: 20 µm. (*J*) Quantifying the relative fluorescence intensity of ILP3 signaling from (*I*). For (*I* and *J*), *n* = 10 and 8 for iEGFP and iE93 mosquitoes, respectively. Statistical significance of two-group comparisons in (*B*, *D*, and *J*) was determined by a two-tailed unpaired *t* test, *P < 0.05. Differences between groups in (*F* and *H*) were indicated by different letters using one-way ANOVA with Tukey’s multiple comparisons test, *P* < 0.05. Data represent mean ± SD.

The phosphorylation of FoxO is regulated by its upstream factor Akt, a direct result of insulin responsiveness in the insulin signaling pathway (23, 24). Therefore, we investigated the phosphorylation levels of Akt (p-Akt) in mosquitoes treated with dsE93 and dsEGFP. Western blot analysis showed a significant decrease (69%) in the p-Akt levels in iE93 mosquitoes (Fig. 6 *A* and *B*). Our findings indicate that Akt activity in the insulin signaling pathway is inhibited after *E93* knockdown compared to controls. This led to decreased phosphorylation level of FoxO and increased translocation of FoxO from the cytoplasm to the nucleus in *E93*-depleted mosquitoes. In addition, we determined the levels of phosphorylated GSK3β (p-GSK3β), another downstream target of Akt, to confirm the impact of decreased Akt activity caused by *E93* deficiency. Mosquitoes treated with dsE93 displayed a significant reduction (67%) in p-GSK3β levels relative to the control mosquitoes treated with dsEGFP (Fig. 6 *C* and *D*), further demonstrating that *E93* knockdown contributes to the impairment of insulin signaling.

The dysfunction of insulin signaling results from defects in either insulin production, secretion, or insulin sensitivity (39). To further validate the involvement of E93 in regulating the insulin cascade, we investigated whether exogenous insulin supplementation could rescue the defects in insulin signaling caused by *E93* knockdown in mosquitoes. The Western blot results showed that dsEGFP- and dsE93-treated mosquitoes exhibited increased levels of p-Akt in response to insulin stimulation. Moreover, no difference was observed in the abundance of p-Akt between iE93 and iEGFP mosquitoes upon insulin injection, indicating that insulin supplementation rescues the decrease in Akt phosphorylation caused by *E93* depletion. Insulin-injected iE93 mosquitoes showed a 4.92-fold increase in p-Akt levels compared with their PBS-injected counterparts, whereas iEGFP control mosquitoes showed a 1.94-fold increase (Fig. 6 *E* and *F*). This result indicates that the basal Akt phosphorylation is lower in *E93*-depleted mosquitoes than in controls, consistent with the findings in Fig. 6 *A* and *B*. Subsequently, we determined the phosphorylation state of GSK3β following insulin stimulation. GSK3β phosphorylation in mosquitoes treated with dsE93 and dsEGFP exhibited a similar insulin-responsive pattern to Akt phosphorylation, with 2.77-fold (iE93 mosquitoes) and 1.61-fold (iEGFP mosquitoes) increases, respectively (Fig. 6 *G* and *H*). Thus, we conclude that although the loss of *E93* leads to a defect in the insulin signaling pathway, the sensitivity of iE93 mosquitoes to insulin remains unchanged.

Hence, we raise the possibility that signals upstream of Akt in the insulin pathway might be disturbed after *E93* knockdown. ILPs, secreted from the IPCs, serve as the upstream signal molecules to activate the insulin signaling cascade (40). We assessed the impact of *E93* loss on the expression of eight *ILP* to examine whether E93 modulates the production and release of ILPs. Transcripts of *ILP3*, *ILP4*, *ILP6*, and *ILP8* were significantly reduced in the head of iE93 mosquitoes compared to controls, whereas *ILP2* was increased, and *ILP1, ILP5,* and *ILP7* remained unchanged (*SI Appendix*, Fig. S4*A*). In the fat bodies, except for *ILP7* and *ILP8*, the mRNA expression of *ILP1* to *ILP6* was markedly decreased following *E93* knockdown (*SI Appendix*, Fig. S4*B*). Collectively, *ILP3*, *ILP4*, and *ILP6* exhibited a lower expression both in the head and fat bodies of iE93 mosquitoes. However, the metabolic phenotype observed in *ILP6* knockout mosquitoes was opposite to that of iE93 mosquitoes, suggesting that *ILP6* downregulation does not account for the metabolic effects associated with *E93* depletion (22). Therefore, we tested ILP3 and ILP4 signals in brain IPCs by performing immunofluorescence. Signals of ILP3 (Fig. 6 *I* and *J*), but not ILP4 (*SI Appendix*, Fig. S4 *C* and *D*), were dramatically reduced in the IPCs of iE93 mosquitoes compared to controls. These data suggest that *E93* deficiency acts on IPCs to inhibit the production of ILP3, thereby triggering metabolic reprogramming in iE93 mosquitoes.

### E93 Exerts Dual Regulation on the Transcription of *PEPCK* Genes.

Since the insulin signaling was restored by insulin injection in *E93*-depleted mosquitoes (Fig. 6 *E*–*H*), we wondered whether insulin treatment can normalize the expression of *PEPCK* genes. To address this question, we assessed the expression levels of *PEPCK-1* and *PEPCK-2* following insulin treatment using qPCR. Insulin injection significantly downregulated both the transcripts of *PEPCK-1* and *PEPCK-2* in iEGFP and iE93 mosquitoes compared with their PBS-injected counterparts. However, insulin failed to restore the expression levels of *PEPCK* genes in iE93 mosquitoes to those observed in iEGFP groups (*SI Appendix*, Fig. S5*A*), indicating that E93’s regulation of these genes was not limited to the insulin signaling pathway.

**Fig. 7.**
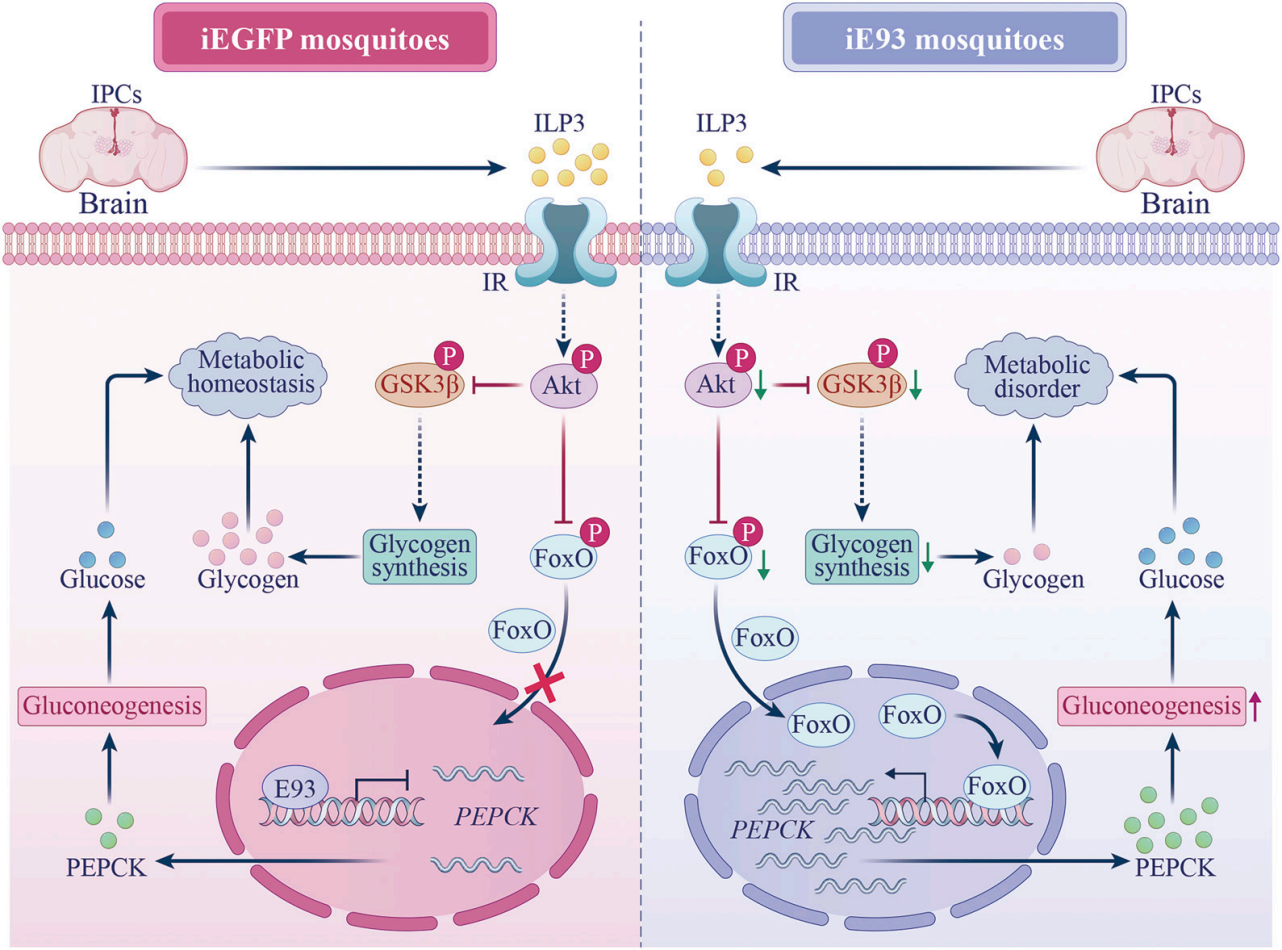
The diagram illustrates the mechanism of E93-mediated metabolic homeostasis in the *A. aegypti* female mosquitoes following a blood meal. In iEGFP mosquitoes, the metabolic homeostasis after blood feeding is maintained through the cooperation of E93’s genomic action and insulin signaling pathway. The transcription factor E93 directly inhibits the expression of *PEPCK*. Simultaneously, IPCs from brains produce sufficient ILP3 in response to blood meal, leading to Akt-mediated phosphorylation of GSK3β and FoxO. These phosphorylation events promote glycogen synthesis and inhibit *PEPCK* expression during gluconeogenesis. Conversely, in iE93 mosquitoes, *E93* deficiency disrupts this regulatory network. Knockdown of *E93* abolishes its direct inhibitory effect on *PEPCK*. Moreover, *E93* deficiency impairs ILP3 production in IPCs, leading to a decrease in Akt/GSK3β phosphorylation and an increase in nuclear FoxO. This results in a decline in glycogen synthesis and an upregulation in the *PEPCK* expression, respectively. Collectively, these disruptions lead to metabolic disorder in iE93 female mosquitoes.

To determine whether E93 can directly regulate the transcription of two *PEPCK* genes, we conducted the dual-luciferase reporter assay. In Aag2 cells, overexpression of the E93 protein significantly inhibited the transcriptional activity of both *PEPCK-1* and *PEPCK-2* compared to the control cells that were cotransfected with reporter vectors and the pAc5.1/V5 empty vector (*SI Appendix*, Fig. S5*B*). This result demonstrates that E93 directly represses the expression of *PEPCK-1* and *PEPCK-2*. Altogether, our observations demonstrate that E93 exerts a dual regulatory effect on the expression of *PEPCK* genes through both insulin-dependent mechanism and direct transcriptional control (Fig. 7).

## Discussion

The E93 factor is the adult specifier because it governs pupal-adult changes (27, 41). In adult mosquitoes, E93 modulates transitions between reproductive cycles (31). A significant portion of genes associated with CM, LM, and amino acid metabolism underwent E93-dependent differential expression at the termination of the vitellogenesis (31). Here, we demonstrated that iE93 mosquitoes exhibited metabolic reprogramming, manifested as the disorder of carbohydrate, lipid, and amino acid levels. These findings reflected the typical hallmark of diabetes, characterized by hyperglycemia and hyperlipidemia, which was similar to that reported in *D. melanogaster* (42). Overall, our study provides a clue for further comprehending the molecular basis of transcription factor E93 and improves our understanding of the tightly coupling between reproduction and metabolism.

Previous studies have established that E93 is involved in chromatin modifications (33, 34). Chromatin accessibility across the whole genome changes dynamically during development, enabling numerous regulatory factors to modulate gene expression cooperatively in response to the developmental cues (33). Our results imply that changes in the accessible regions of the mosquito genome caused by *E93* knockdown either recruit or hinder multiple regulatory factors from accessing their target DNA sequences, thereby affecting the differential transcriptional outcomes. Likewise, during wing development of *D. melanogaster*, E93 was required for the changes in chromatin accessibility, which was correlated with the activity of different enhancers (34). Integrative analysis of the RNA-seq and ATAC-seq data demonstrated that *PEPCK* genes exhibited increased mRNA abundance and enhanced chromatin accessibility in their promoter regions following *E93* knockdown. Loss of *E93* could lead to the recruitment of specific regulatory factors to the regulatory region of *PEPCK* genes, thus activating their expression. Consistent with this hypothesis, we found that *E93* deficiency promotes the nuclear translocation of FoxO, which in turn enhances *PEPCK* transcription, resulting in hyperglycemia.

An analysis of the RNA-seq data and ATAC-seq data revealed that only a small subset of genes was simultaneously altered in both the mRNA abundance and chromatin accessibility. In contrast, many genes exhibited differential expressions regardless of the chromatin accessibility. This suggests that the regulatory mechanism of E93 on gene expression involves various factors beyond the modulation of chromatin accessibility. Based on the sequence and structural information of E93 (43), *A. aegypti* E93 protein contains two conserved Pipsqueak-type helix–turn–helix DNA binding motifs (HTH-Psq), the nuclear receptor interaction motif (LXXLL), and the corepressor C-terminal-binding protein interaction motif (PXDLS). This suggests that E93 could perform its biological function through the HTH-Psq motif or the LXXLL/PXDLS motif. Our luciferase activity assay demonstrated that E93 could act as a transcriptional repressor to directly inhibit *PEPCK* expression. In *B. mori*, E93 was essential for inducing *Atg1* expression by binding two HTH-Psq domains to GAGA-containing motifs in its promoter (43). These findings show that E93 can serve as both an activator and a repressor through its DNA-binding domain. Additionally, *B. mori* E93 was bound to the EcR-USP complex through physical association with USP via the LLQHLL interacting motif to weaken its transcriptional activity (43), further supporting the functional role of the LXXLL/PXDLS motif in E93. In the future, more attention should be paid to the unknown mechanism downstream of E93 regulation.

iE93 mosquitoes displayed the opposite phenotype regarding the lipids and glycogen, with a notable increase in lipid levels and a significant decrease in glycogen contents. Consistently, neuron-specific *E93* knockdown in male *D. melanogaster* showed a TAG and glycogen phenotype like that in *E93*-depleted mosquitoes (29). Conversely, *E93* depletion reduced lipid accumulation in the fat body of female *T. castaneum* and increased glycogen levels in female *D. melanogaster* (28, 29). These findings suggest that E93-mediated differential metabolic phenotypes in different insect species may contribute to the variations in gene regulatory networks and endocrine environments. Mechanistically, E93 has been demonstrated to be one of the primary genes that respond to 20E in various insects (31, 43). The steroid signal is essential in coordinating adult *D. melanogaster* metabolism through E93 and a neuropeptide signaling (29). In *A. aegypti*, lipid and glycogen were significantly accumulated in the absence of 20E receptor *EcR* (4, 5). This finding contrasted with the glycogen phenotype observed in *E93*-depleted mosquitoes, indicating that E93 regulates glycogen levels independently of the 20E/EcR signaling cascades in *A. aegypti*.

In mosquitoes, the absence of *E93* reduced Akt phosphorylation, which in turn promoted GSK3β activity and facilitated the nuclear translocation of FoxO. These results suggest the inactivation of the IIS signaling pathway, which explains the diabetes-like phenotype observed in iE93 mosquitoes. According to extensive research in mammals, diabetes typically results from either a failure of the pancreas to produce enough insulin or a reduction in insulin sensitivity, also known as insulin resistance (39). In this work, insulin rescue experiments combined with mosquito brain immunostaining have revealed that the metabolic disorder observed in iE93 mosquitoes was primarily caused by insufficient ILP3 production in mosquito brains, rather than alterations in insulin sensitivity. Furthermore, these findings suggest that ILP3 produced by IPCs, rather than other ILPs, is responsible for activating IIS signaling during the vitellogenic phase of mosquitoes. Previous studies in *A. aegypti* mosquitoes verified that blood feeding triggered the release of ILP3 from brain neurosecretory cells, which bound to insulin receptor with high affinity (44), suggesting the importance of ILP3 in activating the IIS pathway. Mblk-1, a homolog of E93, was found in the mushroom bodies of the *Apis mellifera* brain (45). Neuron-specific expression of E93 was essential for metabolism (29). Moreover, previous studies have reported that E93 exhibited the highest expression in the mosquito midgut after a blood meal (46). Therefore, we hypothesize that E93 acts through the gut–brain axis to initiate the production of ILP3, thereby activating IIS signaling in response to blood feeding. In *D. melanogaster*, a secretory lipase, Vaha, was identified as being synthesized in the midgut and transported to the brain, where it mediates gut–brain communication to regulate ILP release (47).

In summary, we investigated the molecular mechanism of metabolic regulation by E93 during the gonadotrophic cycle, which deepens our understanding of mosquitoes’ reproductive physiology. The present study uncovers that E93 regulates metabolism within the mosquito’s fat bodies through both the IIS signaling cascade and direct transcriptional repression of *PEPCK* expression (Fig. 7). Notably, although we propose that the defects in lipid and amino acid metabolism are caused by the dysregulation of insulin signaling, the detailed regulatory mechanism still needs to be further explored in subsequent studies. Given that the functional domains of the E93 protein are conserved across insect species, the mechanism revealed here may also provide valuable clues for studying the metabolic regulation of E93 in other insects.

## Materials and Methods

The detailed description of materials and methods is shown in *SI Appendix*, *SI Materials and Methods*. RNAi, qPCR, GC-MS, staining, and quantification of glycogen and LDs (4, 5), ATAC-seq, dual-luciferase reporter assay, insulin tolerance test (48), immunofluorescence, EMSA, and Western blot were performed. Primers used here are shown in *SI Appendix*, Table S4. Probes for EMSA are shown in *SI Appendix*, Table S5.

## Supplementary Material

Appendix 01 (PDF)

Dataset S01 (XLSX)

Dataset S02 (XLSX)

Dataset S03 (XLSX)

Dataset S04 (XLSX)

## Data Availability

The high-throughput sequencing raw data used in this study have been deposited in the Genome Sequence Archive (49) at the National Genomics Data Center (50), China National Center for Biotechnology Information/Beijing Institute of Genomics, Chinese Academy of Sciences (GSA: CRA024364, CRA024365), and are publicly accessible at https://ngdc.cncb.ac.cn/gsa. All other data are included in the manuscript and/or *SI Appendix*.
