# Supplementary material for: Genomic and insulin-mediated control of metabolic homeostasis by the mosquito ecdysone-induced gene E93
Source: Proc Natl Acad Sci U S A. 2025 Oct 29;122(44):e2511572122. doi: 10.1073/pnas.2511572122 (PMC12595457; doi:10.1073/pnas.2511572122)
Supplement: Supplementary file 1 — Appendix 01 (PDF) [file pnas.2511572122.sapp.pdf]

Supplementary Information for

Genomic and insulin-mediated control of metabolic homeostasis by the mosquito ecdysone-induced gene E93

Xueli Wang, Danqian Geng, Kai Shi, Qi Qi, Xiangyang Lyu, Xiaomei Sun, Alexander S. Raikhel, and Zhen Zou

Alexander S. Raikhel

E-mail: alexander.raikhel@ucr.edu

Zhen Zou

E-mail: zouzhen@ioz.ac.cn

This PDF file includes:

SI Materials and Methods

SI References

Figures S1 to S5

Tables S1 to S5

Other supporting materials for this manuscript include the following:

Datasets S1 to S4

SI Materials and Methods

Mosquito maintenance. The Liverpool strain of *A. aegypti* mosquitoes were reared in an incubator under a 12:12 light: dark cycle at 28 ± 2°C with 80% humidity, following previously described methods (4, 5). Adults were sustained with 10% sugar solution and water until they were offered a blood meal. All experiments involving vertebrate animals were conducted in accordance with the guidelines and approval of the Animal Care and Use Committees of the Chinese Academy of Sciences, Institute of Zoology (IOZ20190062).

RNAi. The dsRNA targeting *E93* and *FoxO* was synthesized according to the protocols provided by the T7 RiboMAX Express RNAi system (Promega). As a control, dsRNA for *enhanced green fluorescent protein* (*EGFP*) was generated using its coding sequence. Approximately 1-1.5 μg of dsRNA was injected into the thorax of mosquitoes that emerged within 24 h using a microinjector (World Precision Instruments, USA). Three days post-injection, the injected mosquitoes were provided with a blood meal. Then, mosquito samples were collected at 36 h PBM for examination. The primers used for dsRNA synthesis are listed in Table S4.

qPCR. Total RNA was isolated from the fat bodies of 10 mosquitoes using TRIzol reagent (Invitrogen). cDNA was synthesized according to the manufacturer's protocols of the *Evo M-MLV* RT Mix Kit with gDNA Clean for qPCR (Accurate Biology), and qPCR was then carried out using SYBR Green SuperReal PreMix Plus (Tiangen) on the Applied Biosystems StepOnePlus Real-Time System. Primers used in the qPCR are listed in Table S4.

GC-MS. To detect free fatty acids in the fat bodies of iEGFP and iE93 mosquitoes, samples were collected at 36 h PBM. Fifteen fat bodies from each group were homogenized in 1 ml of a solution comprising 2% H_2SO_4 and 98% methanol. Afterwards, the mixture was incubated at 80°C for one hour. Following the addition of 300 μl of hexane and 1.5 ml of H_2O, centrifugation was performed to extract the fatty

acid methyl esters into the hexane layer. The organic phase was then transferred to autosampler vials for GC-MS analysis (4).

Fat bodies were ground in 400 µl of an ice-cold methanol: ethanol: chloroform (8:1:1) solution and incubated at -20℃ for one hour to determine other small molecule metabolites. After centrifugation, the supernatant was vacuum-dried and resuspended in 40 µl of a saturated solution of O-methoxylamine hydrochloride in pyridine. Next, the mixtures were incubated at 37°C for one hour, after which 50 µl of MSTFA reagent were added. These mixtures were then incubated at 37°C for a further 30 minutes with shaking and diluted with hexane. Finally, the supernatant was transferred to autosampler vials for GC-MS analysis. An Agilent Technologies 6890N GC-5973N mass selective detector equipped with an HP-5MS column was used in the GC-MS analysis as described previously (5).

Glycogen and lipid droplet staining. Staining was conducted as previously reported to visually analyze the glycogen and lipid droplet content in the fat bodies of iEGFP and iE93 mosquitoes at 36 h PBM (4, 5). The fat bodies were fixed in 4% paraformaldehyde (PFA) for glycogen staining at 4°C overnight. The fixed samples underwent dehydration, clearing, impregnation with paraffin, embedding, and were then sectioned into 5-µm slices, which were stained using the Periodic Acid Schiff (PAS) staining kit (Solarbio). The sections were observed under the Leica Versa200 system. Image processing and analysis were conducted using the HALO software. For lipid droplet staining, the dissected fat bodies were washed with PBS and stained with Nile Red (Sigma) for two hours. After washing, fat bodies were mounted on a glass slide and visualized under a Leica STELLARIS 5 confocal microscope system.

Glycogen and TAG quantification. The glycogen and TAG contents were detected as previously described (4, 5). To quantify glycogen, five fat bodies per replicate were homogenized in PBS, and then incubated at 70℃ for 5 minutes. The heat-treated lysates were diluted with PBS accordingly and centrifuged at maximum speed. The supernatant was then incubated with the glucose reagent (Sigma) in the presence or

absence of amyloglucosidase (Sigma). Supernatant treated with PBS served as the blank control. Following a 30-minute incubation at 37°C, the reactions were terminated with 12 N H_2SO_4. For the quantification of TAG, five fat bodies per replicate were homogenized in PBST (0.5% Tween 20) and incubated at 70°C for 5 min. The heat-treated samples were then incubated with PBST or triglyceride reagent (Sigma) at 37°C for 30 minutes. Following centrifugation, the supernatant was incubated with a free glycerol reagent (Sigma) at 37°C for 5 minutes. The absorbance for these two detections was measured in a 96-well plate using a SpectraMax Plus 384 spectrophotometer with a wavelength of 540 nm.

ATAC-seq. Fat bodies harvested at 36 h PBM were rapidly frozen in liquid nitrogen and sent to Biomarker Technologies (www.biomarker.com.cn) for tissue processing and ATAC-seq library construction. Following DNA purification, the resulting libraries were sequenced on the Illumina NovaSeq 6000 platform. Bioinformatic analysis of the generated data were performed utilizing the BMKCloud platform (www.biocloud.net).

Dual-luciferase reporter assay. The coding region of *FoxO* and *E93* was amplified and cloned into the pAc5.1/V5 vector (Thermo Scientific). The promoters of *PEPCK-1* (AAEL000006) and *PEPCK-2* (AAEL000080) were amplified and inserted into the luciferase reporter vector pGL4.10 (Promega) to generate the recombinant reporter vectors pGL4.10-PEPCK-1$^{-1727\text{ to }-1}$ and pGL4.10-PEPCK-2 $^{-1290\text{ to }-1}$. The constructs were then co-transfected into Aag2 cells with pAc5.1/V5-FoxO or pAc5.1/V5-E93 using FuGENE® 6 transfection reagent (Promega). Cells transfected with pAc5.1/V5 empty vector or reporter vectors alone served as controls. In all the transfection groups, pGL4.73 vector (Promega) containing the *Renilla* luciferase (*Rluc*) gene and an SV40 early enhancer/promoter was employed as an internal control to normalize transfection efficiency. Luciferase activity assays were performed according to the Dual-luciferase Reporter Assay System (Promega) protocol and measured using a PerkinElmer EnSight microplate reader with Kaleido 3.0 data acquisition and analysis software.

Nuclear and cytoplasmic protein extraction. To detect the distribution of FoxO protein, nuclear and cytoplasmic proteins were extracted from the fat bodies of

mosquitoes at 36 h PBM, following the instructions of NE-PER Nuclear and Cytoplasmic Extraction Reagents kit (Pierce). The protein extracts were then utilized to perform a Western blot. The loading control for the nuclear and cytoplasmic proteins was a H3 monoclonal antibody (Easybio, BE3015) and a monoclonal β-tubulin antibody (Absin, abs137976), respectively.

Western blotting. Proteins from the fat bodies of mosquitoes were extracted using the ice-cold RIPA lysis buffer (CWBIO), supplemented with 1 × protease inhibitor cocktail (Thermo Scientific) and 1 × phosphatase inhibitor cocktail (Roche). Protein samples were separated using SDS-PAGE and transferred to a polyvinylidene fluoride (PVDF) membrane (Merck Millipore). Later, the membranes were blocked and incubated with the corresponding primary antibodies and horseradish peroxidase (HRP)-conjugated secondary antibodies (Easybio). Finally, the bands were visualized using X-ray films in a darkroom after exposure to the SuperSignal^{TM} West Pico PLUS Chemiluminescent Substrate (Thermo Scientific). Band intensities from the blotting were quantified using ImageJ software. Western blots were performed using the antibodies against V5-tag (Invitrogen, R96025), p-Akt (Cell Signaling Technology, 9271S), p-GSK3β (Cell Signaling Technology, 9336S), and FoxO (Produced and purified by GenScript). Tubulin and H3 antibodies served as the loading controls.

EMSA. NEs were obtained from the whole bodies of female mosquitoes using NE-PER Nuclear and Cytoplasmic Extraction Reagents kit (Pierce). EMSA was conducted according to the guidelines in the LightShift Chemiluminescent EMSA Kit (Thermo Fisher Scientific). For differential experimental purposes, mosquitoes that emerged at 72 h PE or treated with dsEGFP/dsFoxO were used. The 40 fmol biotin-labeled probes were incubated with NEs for 20 min and separated with a 5% polyacrylamide gel. The growing amounts of unlabeled-competitive probes (400 fmol, 2000 fmol, and 4000 fmol) and the unlabeled base-mutated probes (4000 fmol) were used in the competitive assays. To determine the existence of FoxO protein in the binding complexes, anti-FoxO polyclonal antibodies were pre-incubated with nuclear extracts before the

addition of biotin-labelled probes. Preincubation with anti-IgG antibody served as the control. Probes used here are shown in Table S5.

Insulin tolerance test. Previous reports have proved the effectiveness of human insulin in activating the IIS pathway in *Anopheles stephensi* (48). Thus, 10 μg/μl recombinant human insulin (Procell) was injected into the thorax of iEGFP and iE93 mosquitoes at 36 h PBM to perform the insulin tolerance test. The counterpart mosquitoes, which were injected with PBS, served as the controls. Following a 30-minute recovery period, their fat bodies were collected for protein extraction and Western blot analysis.

Immunofluorescence. To identify the subcellular localization of FoxO, mosquito fat bodies were dissected and then fixed in 4% PFA at RT for 10 minutes. After permeabilizing and blocking, tissues were sequentially incubated with an anti-FoxO polyclonal antibody and Alexa Fluor 546 secondary antibody (Thermo Scientific). Nucleus staining was performed using Hoechst 33342 (Thermo Scientific). Before imaging, the samples were mounted on glass slides with ProLongTM Glass Antifade Mountant (Invitrogen).

For the immunofluorescence detection of ILP3 and ILP4 in IPCs, mosquito brains were dissected and fixed in 2% PFA. After blocking with 5% goat serum, samples were incubated with anti-ILP3/ILP4 polyclonal antibodies and Alexa Fluor 488 secondary antibody (Thermo Scientific) at 4℃. Following washing, brains were fixed with 4% PFA for at least 4 hours at RT. Then, samples were dehydrated through an ethanol series (30%, 50%, 70%, 95%, 100%) and cleared in xylene. Finally, the brain samples were mounted in DPX and allowed to dry for 48 hours before imaging. The neuronal marker Cy3-AffiniPure Goat Anti-HRP polyclonal antibody (Jackson) was used to counterstain the brain neurons. The images were observed using a Leica STELLARIS 5 confocal microscope system. The fluorescence intensity of each image was analyzed using the ImageJ software.

Statistical analysis. Statistical analyses for comparisons between two groups were performed using either a two-tailed unpaired *t* test, with or without Welch's correction,

or a Mann-Whitney *U* test, depending on the data distribution and variance. One-way ANOVA with Tukey's or Dunnett's T3 multiple comparisons was utilized for comparisons involving more than two groups. All statistical analyses were conducted with GraphPad Prism 10 software. For ATAC-seq analysis, the R package of DiffBind was employed to identify the differential peaks. FC >1.5 or < 0.67 and FDR < 0.05 were set as the criteria for DARs. GO enrichment analysis was conducted using the DAVID web server, with a significance threshold set at $P < 0.05$, as determined by Fisher's Exact test.

SI References

4. X. Wang et al., Hormone and receptor interplay in the regulation of mosquito lipid metabolism. *Proc. Natl. Acad. Sci. U. S. A*. **114**, E2709-E2718 (2017).

5. Y. Hou et al., Temporal coordination of carbohydrate metabolism during mosquito reproduction. *PLoS. Genet*. **11**, e1005309 (2015).

48. M. A. Kang, T. M. Mott, E. C. Tapley, E. E. Lewis, S. Luckhart, Insulin regulates aging and oxidative stress in *Anopheles stephensi*. *J Exp Biol*. **211**, 741-748 (2008).

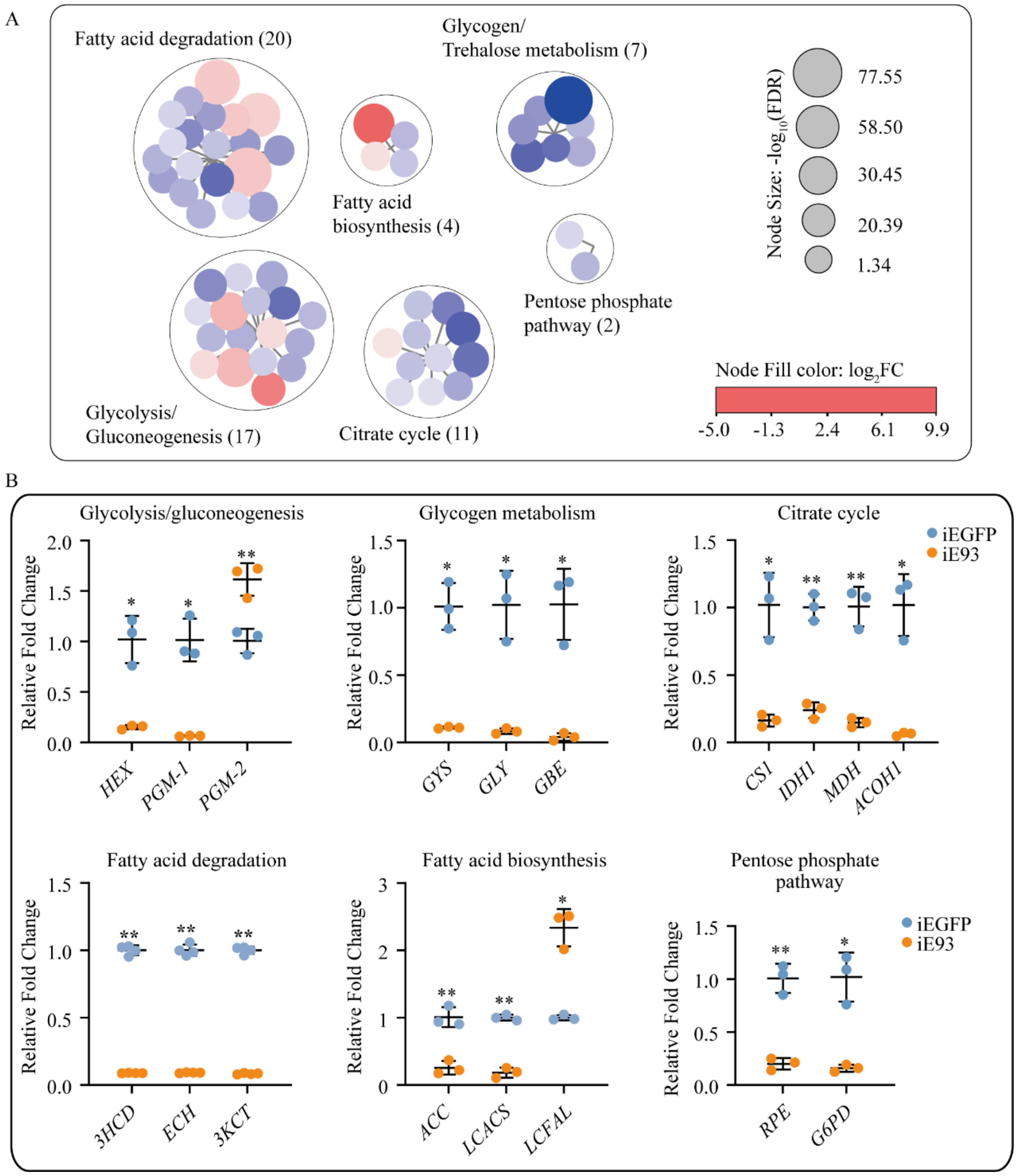

Fig. S1. *E93* knockdown regulates the expression of genes related to CM and LM. (*A*) RNA-seq data revealed differential gene expression associated with CM and LM. Blue, downregulated. Red, up-regulated. (*B*) qPCR analysis validated the RNA-seq data for representative genes in the corresponding signaling pathway shown in (*A*). *HEX*, *Hexokinase*. *PGM*, *Phosphoglucomutase*. *GSY*, *Glycogen synthase*. *GLY*, *Glycogen phosphorylase*. *GBE*, *Glycogen branching enzyme*. *CS*, *Citrate synthase*. *IDH*, *Isocitrate dehydrogenase*. *MDH*, *Malate dehydrogenase*. *ACOH*, *Aconitate hydratase*. *3HCD*, *3-hydroxyacyl-coa dehydrogenase*. *ECH*, *Enoyl-CoA hydratase*. *3KCT*, *3-ketoacyl-coa thiolase*. *ACC*, *Acetyl-CoA carboxylase*. *LCFAS*, *Long-chain fatty acid*

CoA synthetase. *LCFAL*, *Long-chain-fatty-acid-CoA ligase*. *RPE*, *Ribulose-phosphate 3-epimerase*. *G6PD*, *Glucose-6-phosphate dehydrogenase*. A two-tailed unpaired *t* test with Welch's correction was used to do the statistical analysis. Data are represented as mean ± SD. * $P < 0.05$, ** $P < 0.01$.

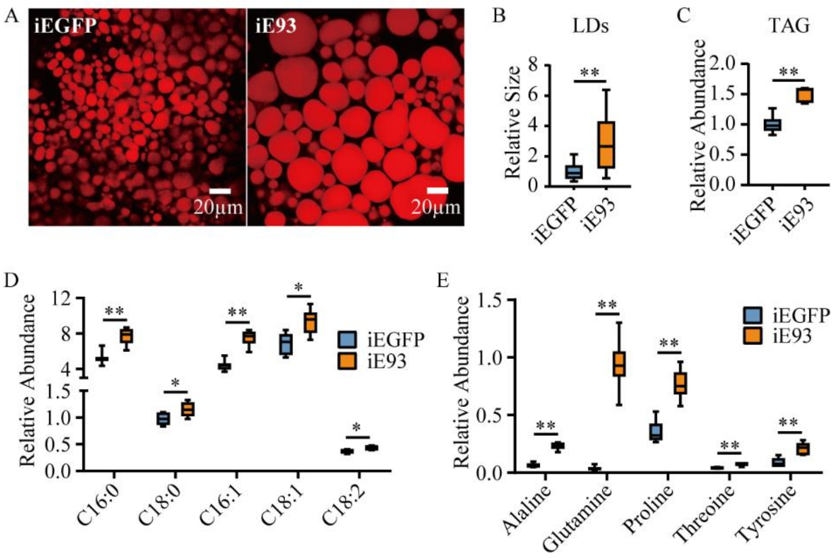

Fig. S2. *E93* deficiency disrupts lipid and amino acid metabolism. (*A*) Lipid droplets in iEGFP and iE93 mosquitoes were visualized by Nile Red staining. Scale bar: 20 µm. (*B*) The relative size of LDs shown in (*A*) was measured using ImageJ software. (*C*) Comparison of endogenous TAG content between iEGFP (n = 6) and iE93 (n = 5) mosquitoes. (*D* and *E*) The relative amount of free fatty acids (*D*) and amino acids (*E*) was determined by GC-MS analysis. $n \geq 5$. Box plots in (*B*-*E*) display the median values, lower and upper quartiles. Error bars show the minimum and maximum values. Two-tailed unpaired *t* test with Welch's correction was used for the statistical analysis of C18:0, and Mann-Whitney *U* test was used for comparison of the other metabolites. * $P < 0.05$, ** $P < 0.01$.

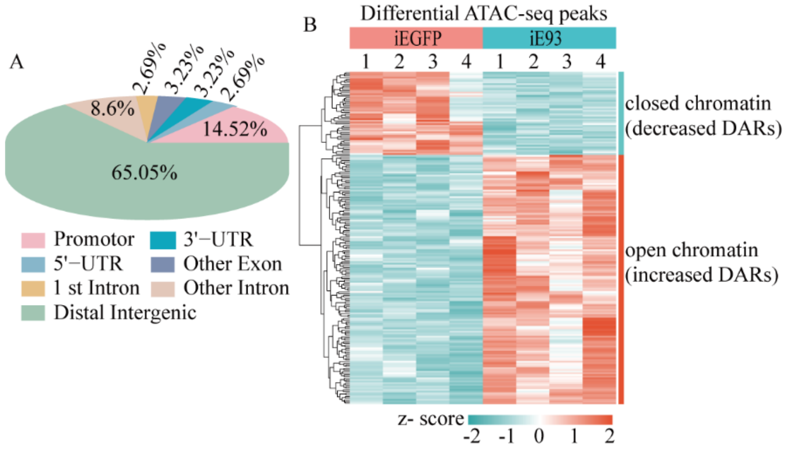

Fig. S3. Distribution and chromatin status of the peaks identified in the ATAC-seq analysis. (*A*) The distribution and proportion of differential peaks across the genome. (*B*) Heatmap showing DARs in iEGFP and iE93 mosquitoes. All data were normalized using z-score, with a z-score < 0 indicating a closed chromatin region (decreased DARs), while a z-score > 0 indicating an open chromatin region (increased DARs).

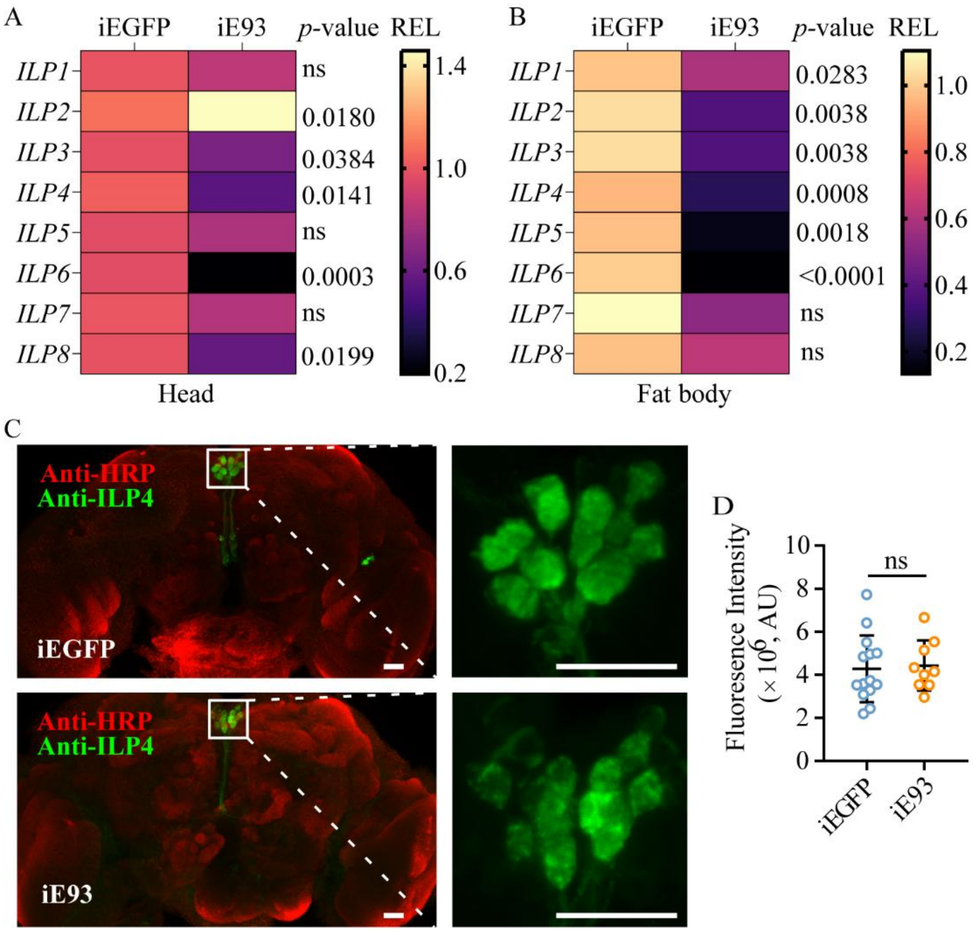

Fig. S4. Examination of the expression of *ILP* genes in mosquito tissues and the visualization of ILP4 in mosquito brains. The expression abundance of eight *ILP* genes in head (*A*) and fat body (*B*) was determined by qPCR. REL, relative expression levels. Two-tailed unpaired *t* test with Welch's correction was used for the statistical analysis in (*A*) and (*B*), except for *ILP7* expression in the fat bodies, which was analyzed using the Mann-Whitney *U* test. $n \geq 3$. (*C*) Immunofluorescence of the endogenous ILP4 (green) in iEGFP and iE93 mosquitoes' brains. Neural cells (red) in brain were stained by anti-HRP antibody. Area in the white boxes is magnified at the right of each group. Scale bar: 20 µm. n = 14 and 9 for iEGFP and iE93 mosquitoes, respectively. (*D*) Quantification of the relative fluorescence intensity of ILP4 signaling from (*C*). A two-tailed unpaired *t* test was used in the statistical analysis of (*D*). Data represent mean ± SD. ns, not significant.

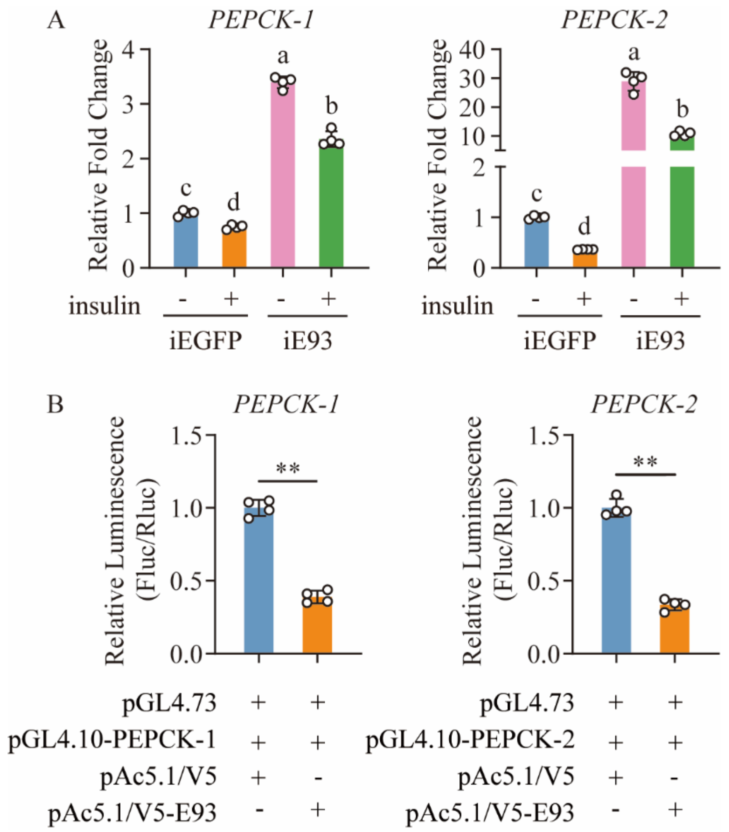

Fig. S5. Genomic action of E93 on the expression of *PEPCK* genes. (*A*) Expression of *PEPCK* genes in iEGFP and iE93 mosquitoes following insulin treatment. Differences between groups were indicated by different letters using one-way ANOVA with Tukey's multiple comparisons test for *PEPCK-1* and Dunnett's T3 test for *PEPCK-2*, $P < 0.05$, Data represent mean ± SD. (*B*) E93 represses the expression of *PEPCK* genes. A dual-luciferase reporter assay detected the effects of overexpressed V5-E93 on *PEPCK* promoters. $n = 4$. Statistical significances were determined by a two-tailed unpaired *t* test. Data represent mean ± SD. ** $P < 0.01$.

Table S1. List of the target genes corresponding to the 150 increased peaks for Sankey dot analysis.

**Description**	**Gene Ratio**	***p*-value**	**GeneID**	**Count**
oxidoreductase activity	0.067	0.017	AAEL003203<br>AAEL007815<br>AAEL005006<br>AAEL017029<br>AAEL000834<br>AAEL004573	6
transcription factor binding	0.022	0.039	AAEL008842<br>AAEL002427	2
phosphoenolpyruvate carboxykinase activity	0.022	0.024	AAEL000080<br>AAEL000006	2
gluconeogenesis	0.022	0.044	AAEL000080<br>AAEL000006	2
fatty acid biosynthetic process	0.034	0.032	AAEL003203<br>AAEL001194<br>AAEL004573	3
single-organism metabolic process	0.124	0.028	AAEL000213<br>AAEL003203<br>AAEL001194<br>AAEL001747<br>AAEL013365<br>AAEL000080<br>AAEL000006<br>AAEL017029<br>AAEL006987<br>AAEL004573<br>AAEL009658	11

Table S2. Information of the genes used for the Sankey dot analysis.

GeneID	Gene description	Abbreviations
AAEL009658	trehalose	TREA
AAEL006987	putative phosphatidate phosphatase	PAP
AAEL013365	lysosomal acid lipase	LAL
AAEL001747	fatty acyl-CoA reductase wat	FAR
AAEL000213	d-amino acid oxidase	DAAO
AAEL001194	fatty acid synthetase	FASN
AAEL000006	phosphoenolpyruvate carboxykinase-1	PEPCK-1
AAEL000080	phosphoenolpyruvate carboxykinase-2	PEPCK-2
AAEL002427	transcription factor IIIB 90 kDa subunit (TFIIIB90)	TFIIIB90
AAEL008842	CREB-regulated transcription coactivator 1	CRTC1
AAEL004573	delta (9)-desaturase 2, putative	Δ9D2
AAEL000834	flavin-containing monooxygenase	FMO
AAEL017029	protein henna isoform X2	ProHenna
AAEL005006	cytochrome P450 6g2	CYP6CD1
AAEL007815	cytochrome P450 4d1-like	CYP4D24
AAEL003203	fatty acid desaturase	FAD

Table S3. Information of the co-upregulated target genes both in the RNA-seq and ATAC-seq for the GO analysis.

Category	Term	Genes	Count	Ratio %	*p*-value
Biological Process	gluconeogenesis	AAEL000080 AAEL000006	2	0.077	0.012
Molecular Function	PEPCK (GTP) activity	AAEL000080 AAEL000006	2	0.077	0.0086
Molecular Function	GTP binding	AAEL000080 AAEL000006 AAEL017301	3	0.115	0.092

Table S4. Primers used in this study.

Gene name	Primers (5'-3')	Primer use
HEX	CTAGATCTGGGCGGTACCAA<br>GTCCCACTGCCAAGCATAAT	qPCR
PGM-1	AAGGACGGAGTGGTTCACAC<br>ATGTCGTAATCTCCGTTCCG	qPCR
PGM-2	TTCATTTCGAGGAAACCCTG<br>TGGAGAACACATGAATCCGA	qPCR
GYS	TGAAAGGTGTGCGGGCGTGT<br>TTGCAACCGCAGTGCCTTCC	qPCR
GLY	TTCATGTGCGCTGCCACCCT<br>TCGAACGATGTGCGAACGGCA	qPCR
GBE	TGATCGTGCGCAAGCAGGAC<br>ATGCCACACGCGCTGCTGAT	qPCR
CS1	AAGGTGGCAACGTGTCCGCT<br>TCCGTTCATACCGGCAGCGA	qPCR
IDH1	TTTGCCGGAGTGCCGGTTGA<br>AGGGCGACGCCATTTCGCTT	qPCR
MDH	ATCCCGCCGTGGCCTTCAAA<br>TGACGTTGGCCGACAGCAGA	qPCR
ACOH1	AAGGCTGGCAGTGCGCTGAA<br>ATGCGAGCGGGATGCGGAAA	qPCR
3HCD	TGGCTACCAGGTCACCATTT<br>AGGCTGTTCAGCTCATCCTG	qPCR
ECH	AAATTACCTGTCGGCTCTGC<br>GCTTCAGGGTTTCGAAGTTG	qPCR
3KCT	GCGTCTTCAAGAGCGAAATC<br>CAAAGATGGCAGCTTGTTCA	qPCR
ACC	ACGCGTTGGCGGAACTGGAA<br>TCGCTCCGGTGTGTCGTGAA	qPCR
LCACS	GAAGCCAAGCCGACGCGTAT<br>TGGCGGTTCCGCTTTGTGCT	qPCR
LCFAL	ACTCGGTGCCGCCAAATCGT<br>ACACTCCGGCAACACCGGAA	qPCR
RPE	AAGGTGCACTGGTTGCGGGA<br>ATTTGCGCCGGCTTTCGCAC	qPCR
G6PD	TTCGGGTGCAACCCGGTGAA<br>AGGCGTCCGGCAGTTTGACA	qPCR

PEPCK-1	TGTCTTCTGGGAAGGAATGG<br>TCACCTTTCTTCCAGGGTTG	qPCR
PEPCK-2	CGGGTACAACTTCGGTGACT<br>ACTTTCCATTGGCATCCTTG	qPCR
ILP1	GCTTCAGTTTCATCCCTTCT<br>AACTCTTGCGGCAGCACTCG	qPCR
ILP2	AAGTCGAGGTCGAAGTGGCT<br>TCTTGCAGCATTCGTCGTAG	qPCR
ILP3	GGTGCTCACCTTGTCGATGC<br>TCGGTGCTGTAGTCTTTGTC	qPCR
ILP4	TTTACTCGAAGCACGACCCT<br>CCTCTACGATGCCTTTCCCT	qPCR
ILP5	ACGATGATTACGGCGGTGGA<br>GCTTTGCGCTTGCTCTTTGC	qPCR
ILP6	ACGACGATCACACCGCCGTT<br>TGCTCTCTTCGTTGGACGCC	qPCR
ILP7	TCGGTATCCTTTCCAGTCGT<br>TTTCGCAGTAAGCGGTGAGT	qPCR
ILP8	TCGAGGGCCATTCTACAAGC<br>TCCGCAACATCACAGACAAC	qPCR
FoxO	CATCTGGAACCGGAATGTCT<br>AACGGTACTGGGCGTAAGTG	qPCR
FoxO	T7-GCTTGGGGTAACCTGTCGTA<br>T7-TGCTAGATCCGGTGGATAGG	dsRNA
E93	T7-TTGGCGTGACAACGCAGCGA<br>T7-TTGTGCAGCACGTCCCGCTT	dsRNA
PEPCK-1	TCAATGGCGTGTGGCAAT<br>GAACACAGCACTCCTCA	Luciferase
PEPCK-2	GACCATGTCTCTTTCAA<br>TTTGACGAGATTCTTAACAC	Luciferase

Table S5. Probes used in EMSA.

**Probe names**	**Probe sequence (5'-3')**
FRE-1 (*PEPCK-1*)	CAGCTCATTTGAAAACAAAAACATT
FRE-2 (*PEPCK-1*)	ACAAACATCGAAAAACAAAAAAAAA
FRE-3 (*PEPCK-1*)	TTAAACAATGTGTTCTATTGTTTAG
FRE-4 (*PEPCK-1*)	TCTAGAATCCAAAAAACAAGTCATTGT
FRE-5 (*PEPCK-1*)	ACTTGGTTTGTTTTTGAGTCATAAA
FRE-6 (*PEPCK-1*)	CACATTTTTGAAAAACAAAACACCG
FRE-7 (*PEPCK-1*)	AAAATAAATGATAAACAAATACGCCTCT
FRE-8 (*PEPCK-1*)	GTGGTTGCGAAAAACAAGTTTAGGAGAC
FRE-9 (*PEPCK-1*)	GCTTTTTGTTGTACACACAAAGCGA
FRE-8-mutated (*PEPCK-1*)	GTGGTTGAACCCCCACCGTTTAGGAGAC
FRE-1 (*PEPCK-2*)	TATGGATGCATGTGTATTGCGGAGATCAG
FRE-2 (*PEPCK-2*)	TAATGAAAATAAAACACATGATTCAATCG

Dataset S1. List of genes associated with LM and CM identified in RNA-seq data.

Dataset S2. List of the differential peaks identified in the ATAC-seq analysis.

Dataset S3. Information about differential peaks in the volcano plot.

Dataset S4. List of uniquely and commonly regulated genes in RNA-seq and ATAC-seq data for the Venn diagram and UpSet plot.
